# Long-term disease interactions amongst surgical patients: a population cohort study

**DOI:** 10.1016/j.bja.2023.04.041

**Published:** 2023-07-01

**Authors:** Alexander J. Fowler, M.A. Hussein Wahedally, Tom E.F. Abbott, John R. Prowle, David A. Cromwell, Rupert M. Pearse

**Affiliations:** 1 School of Medicine and Dentistry, Queen Mary University of London, London, UK; 2 Royal College of Surgeons of England, London, UK; 3 London School of Hygiene and Tropical Medicine, London, UK

**Keywords:** health services research, multi-morbidity, perioperative medicine, surgery, surgical outcomes

## Abstract

**Background:**

The average age of the surgical population continues to increase, as does prevalence of long-term diseases. However, outcomes amongst multi-morbid surgical patients are not well described.

**Methods:**

We included adults undergoing non-obstetric surgical procedures in the English National Health Service between January 2010 and December 2015. Patients could be included multiple times in sequential 90-day procedure spells. Multi-morbidity was defined as presence of two or more long-term diseases identified using a modified Charlson comorbidity index. The primary outcome was 90-day postoperative death. Secondary outcomes included emergency hospital readmission within 90 days. We calculated age- and sex-adjusted odds ratios (OR) with 95% confidence intervals (CI) using logistic regression. We compared the outcomes associated with different disease combinations.

**Results:**

We identified 20 193 659 procedure spells among 13 062 715 individuals aged 57 (standard deviation 19) yr. Multi-morbidity was present among 2 577 049 (12.8%) spells with 195 965 deaths (7.6%), compared with 17 616 610 (88.2%) spells without multi-morbidity with 163 529 deaths (0.9%). Multi-morbidity was present in 1 902 859/16 946 808 (11.2%) elective spells, with 57 663 deaths (2.7%, OR 4.9 [95% CI: 4.9–4.9]), and 674 190/3 246 851 (20.7%) non-elective spells, with 138 302 deaths (20.5%, OR 3.0 [95% CI: 3.0–3.1]). Emergency readmission followed 547 399 (22.0%) spells with multi-morbidity compared with 1 255 526 (7.2%) without. Multi-morbid patients accounted for 57 663/114 783 (50.2%) deaths after elective spells, and 138 302/244 711 (56.5%) after non-elective spells. The rate of death varied five-fold from lowest to highest risk disease pairs.

**Conclusion:**

One in eight patients undergoing surgery have multi-morbidity, accounting for more than half of all postoperative deaths. Disease interactions amongst multi-morbid patients is an important determinant of patient outcome.

**Editor's key points**

• Chronic diseases add to perioperative risk and reduce longer-term disability-free survival.
• This study focussed on the impact of combinations of chronic diseases on 90-day postoperative mortality.
• The commonest triad amongst elective patients was a combination of airways disease, diabetes mellitus and chronic kidney disease.
• Multi-morbidity accounted for half of all postoperative deaths.

The management of people with long-term disease has been highlighted as a key national healthcare priority in the UK.^1^ Around one-quarter of the UK population has two or more long-term diseases, or multi-morbidity,^1^^,^^2^ and the prevalence increases with age and socioeconomic deprivation.^3^^,^^4^ Patients who suffer from multi-morbidity have disproportionately high healthcare utilisation and reduced long-term survival.^3^ However, little is known about the effect of different combinations of multi-morbidity on patient outcomes after surgery.

Each year, more than 5 million surgical procedures are performed in the UK.^5^ The age of patients undergoing surgery in the UK National Health Service (NHS) is increasing, and one in four patients presenting for surgery have chronic disease.^6^^,^^7^ Small studies suggest that surgical patients with multiple chronic diseases (multi-morbidity) have similar outcomes to those without multi-morbidity.^8^ However, larger studies describe a strong association between multi-morbidity and decreased survival, but used data from Medicare in the USA.^9^ Medicare data are not generalisable to the UK given the very different context of healthcare provision in the USA. Studies that have been performed in the UK are small, and limited to only those with cancer or the elderly.^8^, ^9^, ^10^, ^11^ This is particularly important given the increasing rate of multi-morbidity among younger patients, particularly those from underserved groups.^3^

In the context of an ageing population, multi-morbidity is increasingly common in the community but it is not clear how this translates to patients presenting for surgery.^7^ We require a clear description of the prevalence of multi-morbidity in the overall surgical population to fully understand the impact of this problem on surgical systems. Different disease combinations may be associated with different patient outcomes, and it is important to identify these to improve patient care. The aim of this study was to determine the prevalence of multi-morbidity and the associated outcomes among surgical patients, the most frequent disease combinations, and the disease combinations with greatest impact on mortality.

## Methods

We used Hospital Episode Statistics (HES) records to perform a population cohort study of patients having surgery between January 1, 2010 and December 31, 2015 in England. HES captures data related to hospital admissions with linkage infrastructure enabling longitudinal mapping.^12^ Data access was under the existing approval for HES data by NHS Digital's IGARD (DARS-NIC-15335-H0D1F-v5.4); research ethics committee approval of this analysis of pseudonymised data was not required. We applied the Reporting of studies Conducted using Observational Routinely-collected Data (RECORD) reporting guidelines.^13^

### Cohort selection

We included adults (aged ≥18 yr) undergoing surgical procedures defined according to previously described OPCS 4.7 procedure code lists which we previously refined to remove minor and non-surgical procedures.^5^^,^^6^ These codes define procedures typically performed in an operating theatre, or requiring regional or general anaesthesia. We excluded patients undergoing obstetric procedures identified by OPCS 4.7 codes or an admission method code indicating maternity care (2C, 31, 32, 82, 83). The cohort was constructed as a series of 90-day patient ‘procedure spells’. Procedure spells started on the date of the first surgical procedure for each patient. Any procedure within 90 days of this date was assumed to be related to the index procedure and excluded from our analysis. A surgical procedure performed after this 90-day period was included as a new procedure spell. We used this approach, rather than simply including patients' first procedure, to maximise use of the available data, and to capture information about repeat procedures. We modified this approach based on that taken by the PRAiS model.^14^ We determined dates of admission and discharge by deriving continuous in-patient spells, accounting for transfers between NHS hospitals within England.^15^

### Variables

We classified each procedure spell as elective or non-elective (including emergency admissions and transfers) using admission method codes for the first hospital episode. Procedure spells were categorised as in-patient or day-case using patient class. Age is reported in completed years at the start of spell. We grouped OPCS 4.7 codes based on anatomical location as described previously.^16^

### Multiple disease combinations

Multi-morbidity was identified using an algorithm to categorise different combinations of diseases. Individual diseases were defined using an adapted version of the Charlson comorbidity index based on ICD-10 diagnostic codes.^17^ We defined multi-morbidity as the combination of two or more of the 12 conditions (cardiac failure, kidney disease, respiratory disease, stroke, cancer, diabetes mellitus, dementia, paraplegia, liver disease, myocardial infarction, peripheral vascular disease, rheumatological diseases) included within this version of the Charlson comorbidity index. We excluded HIV as this is very rarely captured in HES records. Relevant diagnostic codes were captured from all admitted patient care episodes up to 2 yr before each procedure spell, linked using a patient level pseudonymiser. We implemented a restriction window to ensure that acute diagnoses (such as exacerbations of chronic pulmonary disease) had to be identified in prior episodes and not the index surgical episode. We removed the first diagnostic code (i.e. code DIAG_01) from the recorded during the first episode in the procedure spell, as this typically reflects the indication for surgery. We combined cancer and metastatic cancer into a single variable. We specifically explored dyad, triads, and quads of diseases and how these associated with outcomes.

### Outcome measures

The primary outcome was death within 90 days after the date of surgery. Date of death was captured from Office for National Statistics civil registration death records. This was linked by NHS Digital to civil registration death data using a patient level identifier. Secondary outcome measures were length of hospital stay, and emergency hospital readmission within 90 days of surgery.

### Statistical analysis

Continuous variables are presented as mean (standard deviation) or median (inter-quartile range). Categorical variables are presented as number (percentage). We present crude and adjusted rates of death, 90-day hospital readmission, and length of hospital stay. The crude rate of death used the number of spells as the denominator. The crude rate of hospital readmission used the number of spells where the patient was discharged alive after index surgery as the denominator. Adjustment was performed for age and sex using logistic regression models. We adjusted length of stay using multivariable negative binomial models. We describe disease combinations as dyads (two diseases), triads (three diseases), and quads (four diseases). We extracted all combinations of diseases for all patients, stratified by admission grouping (elective or non-elective). We included only disease combinations with a prevalence of more than one per 2000 procedure spells, which equates to around one spell per month for each acute NHS trust in England.^18^ As each multi-morbid individual may have multiple disease combinations, each patient may be represented more than once in some tables. We present data for the most common dyads, triads, and quads, and those associated with the greatest rate of death. Adjusted odds ratios (ORs) were calculated using a logistic regression model comparing each disease combination to those with no diseases. We took a prior definition of high-risk procedures as those associated with a rate of 90-day death exceeding one in 20 and applied this to disease combinations of two to four diseases.^19^

The relative risk of death for each individual disease present for a multi-morbid patient was calculated by comparing the age-standardised rate of death for each disease combination, to the single disease in combination with any other disease. For example, for chronic kidney disease and diabetes, the risk ratio compares this with the age-adjusted rate of death associated with chronic kidney disease when at least one other disease of any type is also present. We standardised by age directly using ONS mid-year estimates of the population in 2012.^20^

### Sensitivity analysis

To explore if multi-morbid patients underwent higher risk procedures which may confound the association with death, we did a *post hoc* sensitivity analysis. We calculated the age-standardised rate of 90-day death associated with each procedure for all patients in the cohort, stratified by elective or non-elective surgery. Each procedure was identified by three-character OPCS codes. We identified some rare (*n*<50) procedures so used the rate of 90-day death associated with the two-character version of the code. High-risk procedures were defined as those with a crude rate of 90-day death of ≥5%. We present the prevalence of high-risk procedures amongst multi-morbid and non-multi-morbid patients. We included procedure-related rate of death as a term in the overall model of the association between multi-morbidity and death at 90 days. We added the procedure-related rate of death to the logistic regression model as a continuous variable for each dyad comparison to measure how this altered the association with death. To explore potential cluster effects, we created a mixed-effects model in a random sample of 10% of the cohort. Hospital providers were included as a random intercept, and the presence of multi-morbidity as a slope varying with hospital providers (Supplementary methods). We also explored cluster effects in the commonest and riskiest disease dyads. We explored the interaction between age, surgical setting, and multi-morbidity in a series of logistic regressions (Supplementary methods). To explore if inclusion of patients at multiple timepoints influenced our findings, we randomly selected one surgical episode for each patient and repeated our main analysis.

We created a final adjustment model, including age (modelled as restricted cubic spline with five knots), sex, surgical setting (elective or non-elective), procedure-associated harm (modelled as a restricted cubic spline with three knots), and the presence of multi-morbidity. We estimated the population attributable fraction of death associated with multi-morbidity by dividing the number of deaths expected if multi-morbidity were not present in the model by the number of actual deaths.^21^

## Results

### Cohort characteristics

**Fig 1:**
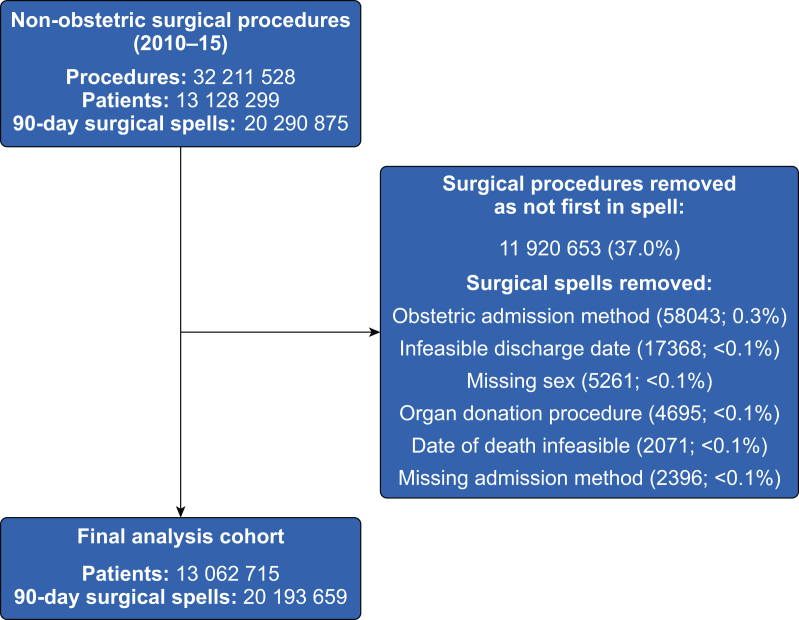
Flow diagram outlining patient selection.

We identified 20 193 659 surgical procedure spells amongst 13 062 715 patients with a mean age of 57.3 (19.2) yr (Table 1, Fig. 1). Some 4 294 138 patients (21.3%) had multiple surgical procedure spells within the dataset. The median number of procedures performed for each 90-day surgical spell was one, some 2 450 089 (12.1%) of patients had more than one procedure during their 90-day spell. Multi-morbidity was present for 2 577 049 spells (12.8%), and the prevalence increased with older ages (Supplementary Fig. S1). We identified 1079 unique disease combinations for between two and four diseases, of which 313 had a prevalence of ≥1 per 2000 spells. Among non-elective surgery spells, 20.7% (674 190 of 3 246 851) featured multi-morbidity compared with 11.2% of elective surgery spells (1 902 859 of 16 946 808) (Supplementary Table S1).

**Table 1:** Characteristics of patients, stratified by presence or absence of multi-morbidity. Data are presented as number (%) unless otherwise stated. CCF, chronic cardiac failure; CKD, chronic kidney disease; DM, diabetes mellitus; IQR, inter-quartile range; MI, myocardial infarction; PVD, peripheral vascular disease; RESP, respiratory diseases; Rheum, rheumatological conditions; SD, standard deviation. ∗Absent and present do not sum to all episodes because over time patients may move between categories of multi-morbidity.

	Patient procedure spells	No multi-morbidity	Multi-morbidity
Spells	20 193 659	17 616 610	2 577 049
Patients∗	13 062 715	12 006 635	1 631 870
Age (yr)			
Median (IQR)	59 (43–73)	56 (40–71)	73 (63–81)
Mean (SD)	57.3 (19.2)	55.3 (19.1)	70.9 (13.5)
Sex, *n* (%)			
Male	9 327 546 (46.2)	7 921 810 (45)	1 405 736 (54.5)
Female	10 866 113 (53.8)	9 694 800 (55)	1 171 313 (45.5)
Admission group, *n* (%)			
Non-elective	3 246 851 (16.1)	2 572 661 (14.6)	674 190 (26.2)
Elective	16 946 808 (83.9)	15 043 949 (85.4)	1 902 859 (73.8)
Inpatient or day-case, *n* (%)			
Day-case	10 960 520 (54.3)	9 988 855 (56.7)	971 665 (37.7)
Inpatient	9 233 139 (45.7)	7 627 755 (43.3)	1 605 384 (62.3)
Ethnicity category, *n* (%)			
White	16 481 581 (81.6)	14 258 195 (80.9)	2 223 386 (86.3)
Unknown	1 877 304 (9.3)	1 740 022 (9.9)	137 282 (5.3)
Asian	740 038 (3.7)	634 519 (3.6)	105 519 (4.1)
Black	393 846 (2)	347 316 (2)	46 530 (1.8)
Other	410 080 (2)	373 973 (2.1)	36 107 (1.4)
Missing	290 810 (1.4)	262 585 (1.5)	28 225 (1.1)
Charlson Comorbidity Index conditions, *n* (%)			
CCF	750 786 (3.7)	150 267 (0.9)	600 519 (23.3)
CKD	962 981 (4.8)	230 235 (1.3)	732 746 (28.4)
RESP	3 050 100 (15.1)	1 816 337 (10.3)	1 233 763 (47.9)
Stroke	494 910 (2.5)	117 116 (0.7)	377 794 (14.7)
Cancer	1 375 777 (6.8)	716 624 (4.1)	659 153 (25.6)
DM	2 248 795 (11.1)	106 7703 (6.1)	1 181 092 (45.8)
Dementia	297 180 (1.5)	99 378 (0.6)	197 802 (7.7)
Paraplegia	140 782 (0.7)	32 767 (0.2)	108 015 (4.2)
Liver	246 580 (1.2)	82 992 (0.5)	163 588 (6.3)
MI	654 286 (3.2)	170 072 (1)	484 214 (18.8)
PVD	724 524 (3.6)	179 409 (1)	545 115 (21.2)
Rheum	522 427 (2.6)	225 245 (1.3)	297 182 (11.5)

### Patient outcomes

Outcomes data are presented in Table 2. The overall rate of death within 90 days after surgery was 1.8%. The overall rate of emergency hospital admission within 90 days after surgery was 9%. Patients with multi-morbidity experienced higher rates of 90-day mortality, longer hospital stays, and higher rates of emergency re-admission to hospital within 90 days after surgery. Among elective surgery spells, the rate of death within 90 days was 2.7% for patients with multi-morbidity compared with 0.4% without (adjusted OR: 4.90 [95% CI: 4.85–4.97], Supplementary Table S2). Among non-elective surgery spells, the rate of death within 90 days was 20.5% for patients with multi-morbidity compared with 4.1% without multi-morbidity (adjusted OR: 3.03 [95% CI: 3.00–3.05]) (Table 2). Patients with multi-morbidity accounted for 50.2% of all deaths after elective surgery (*n*=114 783), and 56.5% of all deaths after non-elective surgery (*n*=244 711).

**Table 2:** Outcomes stratified by the presence or absence of multi-morbidity, presented for non-elective or elective surgery. Data are presented as number (%) of episodes. For death, the denominator is the total number of episodes, for readmissions, the denominator is the total number of eligible episodes. 95% CI, 95% confidence interval; IQR, inter-quartile range; sd, standard deviation. ∗*P*-value comparing multi-morbid with non-multi-morbid patients derived from a χ^2^ test < 0.0001. ^†^*P*-value derived from a Mann–Whitney *U*-test <0.0001. ^‡^Odds ratios adjusted using a logistic regression model including age and sex. ^¶^ Modelled with multivariable, negative binomial model.

	All episodes		Elective surgery		Non-elective surgery	
	No multi-morbidity	Multi-morbidity	No multi-morbidity	Multi-morbidity	No multi-morbidity	Multi-morbidity
*Death within 90 days of surgery*						
Episodes	17 616 610	257 7049	15 043 949	1 902 859	2 572 661	674 190
90-Day deaths	163 529 (0.9)	195 965 (7.6)∗	57 120 (0.4)	57 663 (3)∗	106 409 (4.1)	138 302 (20.5)∗
Adjusted odds ratio (95% CI)^‡^	Ref	4.90 (4.87–4.94)	Ref	4.90 (4.85–4.97)	Ref	3.03 (3.00–3.05)
*Emergency readmission within 90 days of surgery*						
Readmission eligible episodes	17 548 823	248 3777	15 034 351	188 7210	2 514 472	596 567
Readmissions	1 255 526 (7.2)	547 399 (22)∗	872 424 (5.8)	332 410 (17.6)∗	383 102 (15.2)	214 989 (36)∗
Adjusted odds ratio (95% CI)	Ref	3.22 (3.21–3.23)	Ref	3.10 (3.08–3.11)	Ref	2.46 (2.44–2.47)
*Hospital length of stay (days)*						
Mean (sd)	1.9 (8.5)	6.7 (17.1)^†^	1 (4.8)	3.1 (10.7)^†^	7.1 (18.1)	17 (25.5)^†^
Median (IQR)	0 (0–1)	1 (0–6)	0 (0–1)	0 (0–2)	2 (1–6)	8 (2–21)
Length of stay >0 days (%)	6 380 790 (36.2)	1 464 576 (56.8)	4 313 905 (28.7)	836 352 (44)	2 066 885 (80.3)	628 224 (93.2)
Rate ratio for length of stay (95% CI)^¶^	Ref	2.74 (2.73–2.75)	Ref	2.55 (2.54–2.56)	Ref	1.69 (1.69–1.70)

### Commonest disease combinations

The most frequent dyad of diseases across both elective and non-elective surgery was respiratory disease and diabetes mellitus. Among elective spells, the prevalence of this dyad was 2.2% (372 587 of 16 946 808) and among non-elective spells the prevalence was 3.0% (97 179 of 3 246 851). The prevalence of all disease combinations was greater amongst non-elective than elective spells, and for identical disease combinations, patients undergoing non-elective surgery were older (Table 3). Common combinations were all associated with an elevated odds of death; this persisted after adjustment for age and sex. The rate of hospital readmission and length of stay for the commonest dyads is outlined in Supplementary Tables S3 and S4. Diabetes mellitus or respiratory disease were the conditions most frequently present in the common dyads (Table 3). The commonest triad of diseases amongst non-elective patients was cardiac failure and diabetes mellitus and chronic kidney disease (1.1%, 34 616 of 3 246 851). Among elective patients, the commonest triad was airways disease and diabetes mellitus and chronic kidney disease (0.4%, 63 339 of 16 946 808) (Supplementary Table S5). The commonest quad of diseases was cardiac failure and airways disease and diabetes mellitus and chronic kidney disease (Supplementary Table S6).

**Table 3:** Ten commonest disease dyads, stratified by admission group. Death: death within 90 days of surgical procedure. Odds ratios are for death at 90 days compared with patients with no diseases using logistic regression models. Procedure: model included procedure associated harm at 90 days. Age included as a continuous variable without transformation. CCF, chronic cardiac failure; CI, confidence interval; CKD, chronic kidney disease; DM, diabetes mellitus; MI, myocardial infarction; PVD, peripheral vascular disease; RESP, respiratory diseases; Rheum, rheumatological conditions; sd, standard deviation.

Disease combination	*N* (%)	Mean (sd) age	Death within 90 days (%)	Adjusted odds ratio for death (95% CI)	
			Death within 90 days	Sex and age	Sex and age and procedure
*Elective*					
RESP-DM	372 587 (2.2)	67.7 (12.7)	7653 (2.1)	6.70 (6.5–6.88)	5.65 (5.50–5.81)
DM-CKD	244 744 (1.4)	72.0 (12.0)	8089 (3.3)	8.55 (8.32–8.78)	7.16 (6.96–7.37)
RESP-Cancer	220 033 (1.3)	69.3 (12.5)	12 557 (5.7)	18.47 (18.03–18.91)	11.66 (11.37–11.96)
RESP-CKD	177 554 (1.0)	73.7 (12.0)	7255 (4.1)	9.75 (9.48–10.03)	8.04 (7.81–8.28)
DM-Cancer	172 610 (1.0)	71.6 (10.3)	8287 (4.8)	13.30 (12.94–13.67)	9.12 (8.86–9.39)
CCF-RESP	166 468 (1.0)	73.0 (12.0)	8301 (5.0)	12.46 (12.13–12.81)	9.71 (9.44–9.98)
MI-DM	151 214 (0.9)	71.2 (10.6)	4157 (2.7)	7.12 (6.88–7.38)	5.91 (5.70–6.13)
CCF-DM	150 445 (0.9)	72.4 (10.9)	6869 (4.6)	11.76 (11.42–12.10)	9.34 (9.06–9.63)
PVD-DM	142 592 (0.8)	70.0 (11.2)	5139 (3.6)	10.18 (9.85–10.51)	7.87 (7.61–8.14)
PVD-RESP	140 446 (0.8)	70.1 (12.5)	5892 (4.2)	11.79 (11.43–12.15)	8.51 (8.24–8.78)
***Non-elective***					
RESP-DM	97 179 (3.0)	69.4 (14.5)	15 837 (16.3)	4.99 (4.88–5.09)	3.82 (3.73–3.91)
DM-CKD	94 825 (2.9)	73.2 (13.1)	18 436 (19.4)	5.10 (4.99–5.20)	4.22 (4.13–4.31)
CCF-RESP	86 528 (2.7)	75.9 (12.4)	22 377 (25.9)	6.73 (6.59–6.86)	5.32 (5.21–5.43)
CCF-CKD	79 017 (2.4)	78.1 (12.1)	22 471 (28.4)	6.65 (6.52–6.79)	5.73 (5.60–5.85)
CCF-DM	76 460 (2.4)	74.1 (11.8)	16 692 (21.8)	5.80 (5.68–5.93)	4.84 (4.73–4.95)
RESP-CKD	70 928 (2.2)	75.9 (13.0)	17 573 (24.8)	6.13 (6.00–6.27)	4.96 (4.85–5.08)
PVD-DM	68 934 (2.1)	69.9 (12.8)	11 240 (16.3)	4.83 (4.72–4.95)	4.22 (4.11–4.33)
RESP-Cancer	62 043 (1.9)	72.0 (12.4)	22 953 (37.0)	15.19 (14.87–15.51)	7.56 (7.39–7.74)
PVD-RESP	60 240 (1.9)	71.8 (13.5)	12 802 (21.3)	6.15 (6.00–6.29)	4.75 (4.63–4.87)
PVD-CKD	52 762 (1.6)	74.1 (12.9)	12 286 (23.3)	6.03 (5.88–6.18)	5.17 (5.04–5.31)

### Association between disease combinations and outcomes

The dyad with the highest rate of death within 90 days of elective surgery was cardiac failure and liver disease (1339 of 15 041; 8.9%). The dyad with the highest rate of death within 90 days of non-elective surgery was cardiac failure and cancer (10 579 of 27 875; 38.0%) (Supplementary Table S7). Among elective surgical spells, the age-adjusted risk of death at 90 days varied from 1.2% to 8.7% between dyads, and from 6.5% to 34.7% among dyads in non-elective surgical spells (Table 4).

**Table 4:**
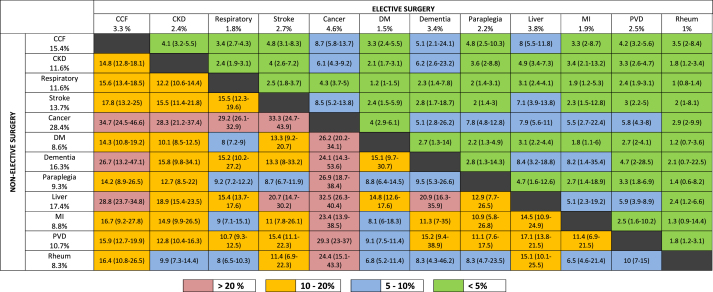
The age-standardised rate of death within 90 days after surgery associated with each disease dyad, stratified by elective or non-elective surgery. Data are presented as percentage (95% confidence interval). Number under each disease is the rate of death at 90 days among multi-morbid patients. The following disease pairs have been included for completeness, but were excluded from the primary analysis as they had an incidence of <1 in 2000 surgical spells: elective surgery; CCF-paraplegia, MI-liver, MI-paraplegia, PVD-paraplegia, CVA-liver, dementia-rheum, dementia-liver, dementia-paraplegia, rheum-liver, rheum-paraplegia, paraplegia-liver, paraplegia-cancer. Emergency surgery; CCF-paraplegia, MI-rheum, MI-liver, MI-paraplegia, PVD-liver, PVD-paraplegia, CVA-rheum, CVA-liver, dementia-rheum, dementia-liver, dementia-paraplegia, rheum-liver, rheum-paraplegia, rheum-cancer, liver-paraplegia, paraplegia-CKD, paraplegia-cancer. CCF, chronic cardiac failure; CKD, chronic kidney disease; DM, diabetes mellitus; MI, myocardial infarction; PVD, peripheral vascular disease; Rheum, rheumatological conditions.

### High-risk disease combinations

Among elective spells, we identified 92 combinations of between two and four diseases with a rate of death exceeding 1 in 20. These are listed in Appendix A. These were present in 837 405 (4.9%) of spells, and 41 789 (5.0%) patients with these combinations died, accounting for 36.4% of all deaths after elective surgery. Cardiac failure, cancer, chronic kidney disease, peripheral vascular disease, and dementia were consistently involved in high-risk combinations. Conversely, paraplegia and rheumatological conditions were rarely a component of high-risk combinations. Among non-elective surgical spells, all combinations exceeded a death rate of one in 20.

### Influence of additional diseases

To identify which diseases promoted poor outcomes in combination with other diseases, we compared all dyads. We present the risk of death associated with each dyad, stratified by elective or non-elective surgery, in Table 4. The relative risk of death associated with different diseases was greater among elective spells. However, the rate of 90-day death associated with different disease dyads was much greater among non-elective spells (8.6–28.4%) than elective spells (1.0–4.6%). Among elective spells, we identified three important patterns (Supplementary Table S8). First, diseases which when added to baseline conditions, are associated with a reduced or unchanged rate of death (respiratory disease, diabetes mellitus, rheumatological conditions). Second, those that increased, but do not double, the relative rate of death for most base conditions (chronic kidney disease, stroke, paraplegia, peripheral vascular disease). Finally, those consistently associated with a doubling in the rate of death (cardiac failure, cancer, liver disease). The influence of dementia was varied, ranging from a relative risk of 1.0 (95% CI: 0.8–5.5) when present in association with cancer, to 4.3 (95% CI: 1.0–9.2) when present in association with myocardial infarction. Cancer when present with other diseases was associated with the greatest rate of death (4.6%). Among patients undergoing non-elective surgery, only cancer was associated with a consistent doubling of the relative risk of death (Supplementary Table S9). All combinations involving cancer were associated with a rate of 90-day death exceeding one in five among patients undergoing non-elective surgery.

### Sensitivity analyses

There was a significant interaction between multi-morbidity and age which was non-linear (Supplementary Fig. S2). We examined if multi-morbid patients more often had high-risk surgery, as a potential confounder of the association with 90-day death. Of 2 577 049 spells associated with multi-morbidity, 589 710 (22.9%) underwent a high-risk surgical procedure compared with 1 161 196 (6.6%) of 17 616 610 spells without multi-morbidity (Supplementary Table S10). When including age-standardised, 90-day rate of death associated with each procedure as a continuous variable in the main model of multi-morbidity, the association was reduced (OR: 3.48 [95% CI: 3.45–3.50]). The effect was similar in our analysis of disease dyads (Table 3). There was a strong interaction between age and procedure associated risk of death (Supplementary Fig. S3). After adjusting for the presence of differing procedure risk, high-risk disease combinations remained strongly associated with elevated death within 90-days (Supplementary Table S7).

When we included provider codes as random intercepts, there was a reduction in the strength of association between multi-morbidity and outcomes (Supplementary Table S2). For example, the OR for 90-day death was 4.66 (95% CI: 4.51–4.83) with random intercept and slope, compared with 4.90 (95% CI: 4.87–4.94) without the intercept or slope. There was a similar effect when we included random intercepts for specified disease dyads (Supplementary Table S11).

Around one in five patients had more than one surgical spell. We randomly selected one surgical spell per patient; the association with 90-day death was unchanged (OR: 4.96 [95% CI: 4.95–5.03]).

When accounting for the presence of multi-morbidity, age, sex, procedural risk, and surgical admission type, the OR for death was 5.58 [5.44–5.73] (Supplementary Table S2). Using this model, we estimate there would be 207 044 deaths assuming multi-morbidity could be eliminated, representing a population attributable fraction of 42%.

## Discussion

**Fig 2:**
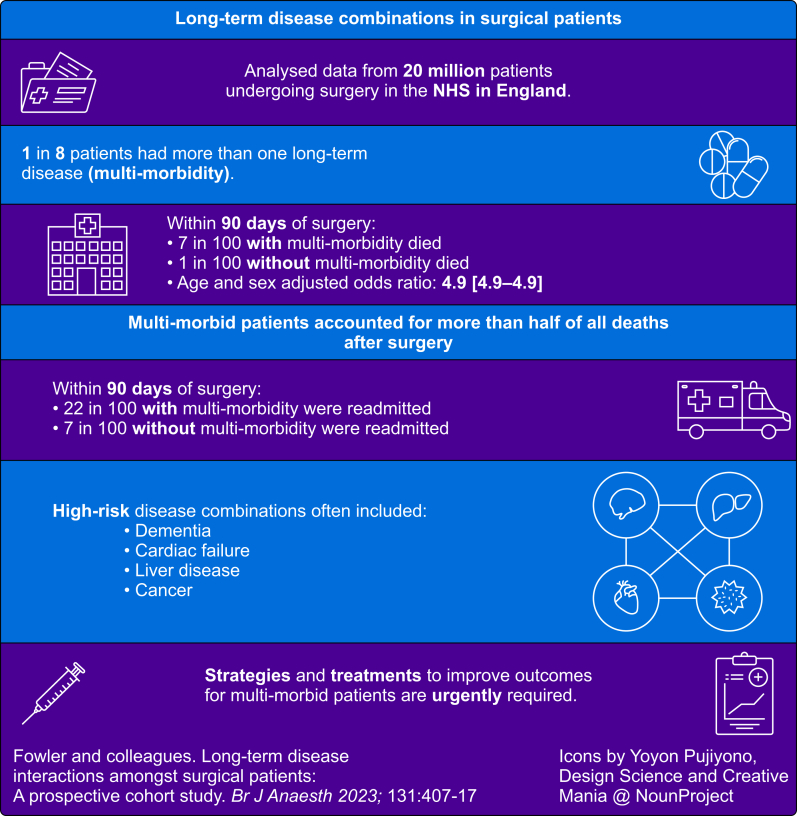
Infographic summarising study findings.

The principal finding of this study is that one in eight patients presenting for surgery have multi-morbidity, of whom one in 13 dies within 90 days (Fig. 2). A high-risk multi-morbidity pattern is present in 5% of elective surgical patients, and these account for more than one-third of all deaths. The prevalence of multi-morbidity, and the associated rate of death is greater among patients undergoing non-elective surgery. The rate of emergency hospital readmission within 90 days of surgery among patients with multi-morbidity exceeds one in five. Patterns of multi-morbidity were complex, and there were not clear disease combinations that defined high-risk patient groups. There was a five-fold variation in risk of death among pairs of diseases, suggesting that counting diseases masks important differences between disease combinations. Half of all deaths after surgery occur among patients with multi-morbidity.

Our findings align with previous studies of multi-morbidity among older surgical patients using large administrative healthcare datasets in other countries. Coagulopathy, electrolyte disturbances, and liver disease were identified in a study of 280 000 patients in the USA.^11^ Our code lists did not include coagulopathy or electrolyte problems, but liver disease was strongly associated with death in many disease combinations. Our findings differ from those of a small multicentre observational study of 413 older patients.^8^ They reported no differences in outcomes between multi-morbid and non-multi-morbid patients. However, three-quarters of their included cohort were multi-morbid, the overall rate of death was high (8.8%), and only 20% had a surgical procedure. These differences may be explained by the larger sample size in our study, variable disease definitions, and our inclusion of only patients having surgical procedures. A US study of older Medicare patients defined lists of disease combinations associated with elevated mortality after general surgery.^9^ They excluded patients with cancer and dementia, and found that one-third of patients had a high-risk combination; these patients experienced a three-fold increase in death.^22^, ^23^, ^24^, ^25^ They termed these combinations qualifying comorbidity sets, which were diverse combinations associated with elevated death.

We found it challenging to identify consistent patterns of multi-morbidity that were associated with a high burden of death and disability. This difficulty has been encountered by researchers exploring multi-morbidity in other domains.^26^ We defined high-risk combinations as those with a rate of death exceeding 5% based on a prior definition of high-risk procedures.^19^ These combinations accounted for one in three deaths after elective surgery. Whereas this approach may be useful to refine multi-morbidity further than simply counting diseases, there were significant overlaps in the diseases present in high- and low-risk combinations. Cancer, cardiac failure, dementia, peripheral vascular disease, and chronic kidney diseases were commonly present in high-risk disease combinations. Among elective patients, chronic kidney disease, cardiac failure, stroke, paraplegia, peripheral vascular disease had an important effect in addition to most base conditions. A doubling in the rate of death was associated with liver disease, cancer, and cardiac failure. Dementia in disease dyads produced different impacts on the risk of death depending on what it was combined with. When combined with cancer, the relative risk of death associated with dementia was slightly increased despite both dementia and cancer being associated with a high rate of death in isolation.^6^ This may reflect patient selection; those with severe dementia and cancer may be offered conservative treatments and not be represented in our cohort. There was a modest reduction in the strength of the effect when accounting for centres in multi-level modelling, suggesting some clustering of effect. This may be because of centralised centres offering specialist services and varying local disease patterns. The five-fold variation in risk of death associated with different disease dyads suggest that counting diseases in comorbidity scales overlooks important between combination differences. Many studies will not have the statistical power required to explore interaction terms and operationalising comorbidity scales with disease interaction weights may be a more practical alternative.

Earlier studies focussed on emergency surgery, we included both elective and non-elective patients.^8^, ^9^, ^10^, ^11^ In our study, patients undergoing non-elective surgery accounted for 16% of spells, but 26% of spells associated with multi-morbidity. It is unclear if this is because multi-morbid patients are more likely to develop acute surgical disease, or if selection criteria differ for elective and non-elective procedures.^27^, ^28^, ^29^ The absolute risk of death associated with multi-morbidity was greatest among non-elective surgical spells, and all disease combinations exceeded 5%. We identified a strong interaction between age and multi-morbidity, which was most pronounced among emergency surgical patients. Certain combinations of long-term diseases were associated with an extremely high risk of death among non-elective surgical patients, such as cardiac failure, liver disease, and cancer which has a risk of death of almost one in two. How best to manage the non-elective, multi-morbid surgical patient is unclear. Recognising they are at high risk of poor outcome is a vital first step informing both shared decision-making and clinical care. Enhanced recovery pathways or provision of dedicated centres may improve outcomes, but the large number of affected patients would prove a significant logistical challenge. The age and sex adjusted odds of death were consistently higher among elective surgical patients. This suggests the influence of multi-morbidity is greater among elective patients who have a lower absolute risk of death. This may be because patients undergoing non-elective surgery reach a ceiling level of risk. We created a final adjustment model and calculated that some 42% of deaths were attributable to multi-morbidity. However, this assumes that multi-morbidity can be perfectly eliminated from the surgical population, and that there is a causal relationship between multi-morbidity and outcomes. While we adjusted for important confounders, there is likely residual unmeasured confounding that means we should be cautious in our interpretation of this fraction.

One potential confounder of the association between multi-morbidity and death after surgery is the nature of the required procedure. For example, patients with a history of cancer may require a more major procedure (such as bowel resection) than those without cancer. We found procedures performed among multi-morbid patients were more often high risk than those performed on non-multi-morbid patients. Some patients with multi-morbidity may not be offered low-risk surgeries given the perceived adverse balance in risk of perioperative complications and patient benefit.^28^ Our cohort consisted only of patients who had procedures, so a more detailed exploration of this was not possible. High-risk surgery may be considered by patients even if there is a high risk of early death, if it may meaningfully improve length and quality of life. We accounted for procedural risk by including the procedure associated rate of death in our adjustment of the association between specific disease combinations and 90-day death. Including procedural risk reduced the association between most disease combinations and 90-day death. However, disease combinations remained strongly associated with death after this adjustment and we identified a statistically and clinically significant interaction between multi-morbidity and procedural risk. An important finding is the high rate of emergency hospital readmission amongst patients with multi-morbidity, which aligns with prior studies.^30^^,^^31^ Around one in five multi-morbid patients experienced an emergency hospital readmission within 90 days of surgery. Hospital readmission is strongly associated with risk of subsequent death.^32^^,^^33^ We observed less variation between elective and non-elective surgical patients in the rate of hospital readmission than in the rate of death. This may be because the background rate of hospital admission among patients with multi-morbidity is already very high.^20^ It may also reflect a deterioration in health after surgery because of complications, worsening of underlying disease, or development of new long-term diseases.^34^ For example, patients who suffer acute kidney injury may go on to develop chronic kidney disease. Identifying and treating patients at the time of complication may prevent progression of their disease.^35^

This analysis has strengths and limitations. We included all patients undergoing surgery performed in, or funded by, the English NHS over a 5-yr period. By using distinct surgical spells, we were able to use information about multiple procedures for each patient which increased the available data size. Some patients may have had repeat procedures beyond our 90-day window for the same indication, however including these is reasonable as they represent new operations. Most prior studies include the first procedure for each patient, which overlooks outcomes of subsequent procedures. Our approach facilitated exploration of relatively rare disease combinations. Some patients may be considered ‘surgically multi-morbid’ if they have had multiple procedures. These patients may have extra considerations, such as the risk of allergic reaction from repeat exposure to neuromuscular blockade agents.^36^^,^^37^ We used robust national death and hospital care records to define outcomes. We used a well-defined code list and process to identify chronic diseases. We removed the first diagnostic code from the index episode when surgery occurred, as this is typically the indication for surgery, meaning that cancer would not be recorded as a chronic disease if it was the indication for surgery. This analysis also has limitations. Studies using diagnostic coding data are prone to misclassification errors, such as misclassifying acute and chronic diseases. We excluded the principal diagnostic code for the index surgical episode to remove the disease predominantly associated with the admission. We also implemented restriction windows to ensure that certain acute diagnoses (e.g. chronic obstructive pulmonary disease exacerbation) had to be reported as a disease on a prior admission only. Diagnostic coding data are also prone to information bias; specifically patients with more severe disease are more likely to be coded. Patients with mild disease may not be identified, and the lack of detailed information about disease severity limited our exploration of this issue. However, the overall prevalence of diseases we identified aligns with prior prospective cohort studies in surgical patients. There are 12 diseases in the modified Charlson index we used, so the possible number of combinations is fairly small compared with other comorbidity indices.^38^ We still found a large number of combinations, which had a complicated relationship with outcomes. Some common diseases such as hypertension and arrhythmias are not included in the Charlson index. Although excluding hypertension will reduce the rate of multi-morbidity, for most patients, hypertension represents an opportunity for targeted prevention rather than a symptomatic disease. We sought to determine the interaction between these diseases, and the Charlson index has been used extensively to explore multi-morbidity in other settings.^38^, ^39^, ^40^ The Charlson index was found to associate more strongly with death than other tools.^41^ Each HES record is limited to 20 diagnostic codes, meaning that there is relatively low coding depth compared with primary care data. For patients with more than 20 diagnoses, how clinical coders decide which to omit from the record is unclear. However, we collated diagnoses from previous hospital admissions to identify important existing disease. This explains the lower prevalence of multiple diseases in our study compared with primary care studies, and it is likely our data represent an underestimate of the prevalence of multi-morbidity.

### Conclusions

One in eight patients presenting for surgery have multi-morbidity. These patients account for more than half of all deaths within 90 days of surgery. More than one in five multi-morbid patients require hospital readmission within 90 days of surgery. Patients requiring non-elective surgery have a higher prevalence of multi-morbidity, and experience worse outcomes. However, we could not identify simple rules that define high-risk multi-morbidity, but counting diseases overlooks important interactions between them. Strategies are required to improve care for multi-morbid patients around the time of surgery. Enhanced recovery after surgery pathways may represent one approach. However, multi-morbid patients may undergo surgeries outside the limited settings where this has demonstrated benefit. Identification of patients with high-risk disease combinations may facilitate stratification to high-risk pathways or enrich randomised trials of perioperative interventions to improve their value.

## Transparency declaration

AF affirms the manuscript is an accurate and transparent account of the study being reported. No important aspects of the study have been omitted.

## Data sharing

HES data were made available by NHS Digital (Copyright© 2021, reused with the permission of NHS Digital. All rights reserved). Approvals for the use of anonymised HES data were obtained as part of the standard approval process. It is not possible to share raw patient-level data without approvals from NHS Digital.

## Patient and public involvement

A patient with lived experience of major surgery was consulted in the design and interpretation of this study.

## Authors’ contributions

Study design: AJF, JP, RP

Data collection and analysis: AJF, HW, DC

Interpretation: all authors

Writing the first draft of the manuscript: AJF

Revision for important intellectual content and approved the final version: all authors

Access to the data and act as guarantors: AJF, DC

## Acknowledgements

This study uses data provided by patients and collected by the NHS as part of their care. We would like to acknowledge and thank all involved in the generation and collection of these data.

## Declarations of interest

AJF holds a National Institute for Health Research Doctoral Research fellowship (DRF-2018-11-ST2-062). MAHW and JP report no relevant conflicts of interest. RP has received honoraria, research grants, or both from Edwards Lifesciences, Intersurgical, and GlaxoSmithKline within the past 5 yr and holds editorial roles with the *British Journal of Anaesthesia* and the *British Journal of Surgery*. TEFA holds a National Institute for Health Research Clinical Lectureship, is a member of the editorial board of the *British Journal of Anaesthesia*, has received research grants from the National Institute for Academic Anaesthesia and Barts Charity, and has received consultancy fees from Merck Sharp & Dohme (MSD) within the past 5 yr unrelated to this work. DC is an associate editor of the *Journal of Health Services Research & Policy*.

## Funding

National Institute for Health and Care Research (NIHR) Doctoral Research Fellowship (DRF-2018-11-ST2-062) to AJF. The funding source had no role in the study design, data collection, analysis, interpretation, or writing the report.
