# Supplementary material for: Long-term disease interactions amongst surgical patients: a population cohort study
Source: Br J Anaesth. 2023 Jul 1;131(2):407–17. doi: 10.1016/j.bja.2023.04.041 (PMC10375505; doi:10.1016/j.bja.2023.04.041)
Supplement: Multimedia component 1 [file mmc1.docx]

**Long-term disease interactions amongst surgical patients: A population cohort study**

**SUPPLEMENTARY FILE**

**Supplementary methods**

We explored potential cluster effects with an exploratory multi-level models including provider codes as random intercepts. Multi-level models account for hierarchical data structures (for example, patients within hospital providers) and generate standard errors that are robust to the potential similarity of individuals within clustering units. Fitting these models were challenging due to model complexity and large data size. We randomly sampled 10% of the cohort and fitted multi-level models with provider as a random intercept and included multi-morbidity as a random slope for each provider. Sex and age were fixed effects. Provider codes were entered at the three-character level which represents organisations i.e., NHS Trusts. We modelled ninety-day death and emergency hospital readmission using multi-level, multivariable logistic regression. We modelled length of stay with a multi-level, multi-variable negative binomial regression. Models were implemented using the GLMMadaptive package in R, which models random effects with the adaptive Gaussian quadrature.

To explore the interaction between age, procedural risk, and death, we created two interaction models. The models included age (modelled with a restricted cubic spline with five knots), procedural risk (modelled with a restricted cubic spline with three knots), sex and surgical setting. We included interactions between multi-morbidity and age (age interaction model), and between multi-morbidity and procedural risk (procedure interaction model). We then estimated the probability of death as age/procedural risk increased. We evaluated the significance of the interaction using ANOVA.

|  | **All episodes** | | **No multi-morbidity** | | **Multi-morbidity** | |
| --- | --- | --- | --- | --- | --- | --- |
|  | **Non-Elective** | **Elective** | **Non-Elective** | **Elective** | **Non-Elective** | **Elective** |
| Episodes | 3246851 | 16946808 | 2572661 | 15043949 | 674190 | 1902859 |
| **Age (years)** | | | | | | |
| Median (IQR) | 58 (38 to 76) | 59 (43 to 72) | 51 (33 to 70) | 57 (42 to 71) | 76 (65 to 84) | 72 (63 to 80) |
| Mean (SD) | 56.4 (22.2) | 57.4 (18.6) | 52.2 (21.6) | 55.8 (18.6) | 72.8 (14.4) | 70.1 (13) |
| **Sex** | | | | | | |
| Male | 1,658,219 (51.1%) | 7,669,327 (45.3%) | 1,305,654 (50.8%) | 6,616,156 (44%) | 352,565 (52.3%) | 1,053,171 (55.3%) |
| Female | 1,588,632 (48.9%) | 9,277,481 (54.7%) | 1,267,007 (49.2%) | 8,427,793 (56%) | 321,625 (47.7%) | 849,688 (44.7%) |
| **Ethnic category** | | | | | | |
| White | 2,686,002 (82.7%) | 13,795,579 (81.4%) | 20,952,86 (81.4%) | 12,162,909 (80.8%) | 590,716 (87.6%) | 1,902,859 |
| Unknown | 227,700 (7%) | 1,649,604 (9.7%) | 198,095 (7.7%) | 1,541,927 (10.2%) | 29,605 (4.4%) | 1,632,670 (85.8%) |
| Asian | 134,093 (4.1%) | 605,945 (3.6%) | 108,311 (4.2%) | 526,208 (3.5%) | 25,782 (3.8%) | 107,677 (5.7%) |
| Black | 70,235 (2.2%) | 323,611 (1.9%) | 579,68 (2.3%) | 289,348 (1.9%) | 12,267 (1.8%) | 79,737 (4.2%) |
| Other | 88,817 (2.7%) | 321,263 (1.9%) | 78,704 (3.1%) | 295,269 (2%) | 10,113 (1.5%) | 34,263 (1.8%) |
| Missing | 40,004 (1.2% | 250,806 (1.5%) | 34,297 (1.3%) | 228,288 (1.5%) | 5,707 (0.8%) | 25,994 (1.4%) |
| **Charlson Comorbidity Index conditions** | | | | | | |
| CCF | 256,088 (7.9%) | 494,698 (2.9%) | 47,654 (1.9%) | 102,613 (0.7%) | 208,434 (30.9%) | 392,085 (20.6%) |
| CKD | 252,642 (7.8%) | 710,339 (4.2%) | 39,484 (1.5%) | 190,751 (1.3%) | 213,158 (31.6%) | 519,588 (27.3%) |
| RESP | 545,813 (16.8%) | 2,504,287 (14.8%) | 252,518 (9.8%) | 1,563,819 (10.4%) | 293,295 (43.5%) | 940,468 (49.4%) |
| Stroke | 171,802 (5.3%) | 323,108 (1.9%) | 32,528 (1.3%) | 84,588 (0.6%) | 139,274 (20.7%) | 238,520 (12.5%) |
| Cancer | 242,707 (7.5%) | 1,133,070 (6.7%) | 94,981 (3.7%) | 621,643 (4.1%) | 147,726 (21.9%) | 511,427 (26.9%) |
| Diabetes | 416,614 (12.8%) | 1,832,181 (10.8%) | 138,880 (5.4%) | 928,823 (6.2%) | 277,734 (41.2%) | 903,358 (47.5%) |
| Dementia | 171,156 (5.3%) | 1,260,24 (0.7%) | 57,883 (2.2%) | 41,495 (0.3%) | 113,273 (16.8%) | 84,529 (4.4%) |
| Hemi-Para | 48,134 (1.5%) | 92,648 (0.5%) | 8,596 (0.3%) | 24,171 (0.2%) | 39,538 (5.9%) | 68,477 (3.6%) |
| Liver | 79,477 (2.4%) | 167,103 (1%) | 25,813 (1%) | 57,179 (0.4%) | 53,664 (8%) | 109,924 (5.8%) |
| MI | 147,717 (4.5%) | 506,569 (3%) | 27,118 (1.1%) | 14,2954 (1%) | 120,599 (17.9%) | 363,615 (19.1%) |
| PVD | 206,638 (6.4%) | 517,886 (3.1%) | 42,708 (1.7%) | 136,701 (0.9%) | 163,930 (24.3%) | 381,185 (20%) |
| Rheum | 92,892 (2.9%) | 429,535 (2.5%) | 28,664 (1.1%) | 196,581 (1.3%) | 64,228 (9.5%) | 232,954 (12.2%) |

**Supplementary table 1. Patient characteristics divided by the presence of multi-morbidity and the admission group of the index procedure.** Data are presented as number (%) unless otherwise stated. CCF; Cardiac failure, RESP; respiratory diseases, CKD; chronic kidney disease, DM; diabetes mellitus, MI; myocardial infarction, PVD; peripheral vascular disease, Rheum; rheumatological conditions

| **Model** | **Variables** | **OR/RR* (95% CI) associated with multi-morbidity** | **Pseudo-R^2^** | **C** |
| --- | --- | --- | --- | --- |
| **Core models** | | | | |
| **90-day death (all)** | Age, sex, multi-morbidity | **4.90 (4.87 – 4.94)** | **0.17** | **0.83** |
| - Elective |  | 4.90 (4.85 – 4.97) | 0.12 | 0.81 |
| - Non-elective |  | 3.03 (3.00 – 3.05) | 0.21 | 0.81 |
| **90-day emergency readmission (all)** | Age, sex, multi-morbidity | **3.22 (3.21 – 3.23)** | **0.05** | **0.63** |
| - Elective |  | 3.10 (3.08 – 3.11) | 0.04 | 0.62 |
| - Non-elective |  | 2.46 (2.44 – 2.47) | 0.07 | 0.65 |
| **Length of hospital stay (all)** | Age, sex, multi-morbidity | **2.74 (2.73 – 2.75)** | **0.02** | **-** |
| - Elective |  | 2.55 (2.54 – 2.56) | 0.02 | - |
| - Non-elective |  | 1.69 (1.69 – 1.70) | 0.03 | - |
| **Sensitivity analyses** | | | | |
| 90-day death including procedure harm | Age, sex, multi-morbidity, age-standardised procedure associated harm | 3.48 (3.45 -3.50) | 0.32 | 0.91 |
| 90-day death, full adjustment model to estimate population attributable fraction | Age, sex, setting, multi-morbidity, age-standardised procedure associated harm* | 5.58 (5.44 – 5.73) | 0.37 | 0.932 |
| 90-day death, restricted to one spell per patient | Age, sex, multi-morbidity | 4.99 (4.95 – 5.03) |  |  |
| **Multilevel models (10% of the cohort)** | | | | |
| **Model** | **Variables** | **OR/RR* (95% CI) associated with multi-morbidity** |  |  |
| 90-day death (all) | Fixed effects: Age, Sex, multi-morbidity  Random intercept: hospital provider  Random slope: multi-morbidity by provider | 4.66 (4.51 – 4.83) |  |  |
| 90-day emergency readmission (all) |  | 3.01 (2.99 – 3.06) |  |  |
| Hospital length of stay (all) |  | 2.48 (2.32 – 2.65) |  |  |

**Supplementary Table 2 Summary of multi-variable regression models. Death and emergency readmission were modelled with logistic regression, length of stay was modelled with negative binomial regression.** *Odds ratio for 90-day death and emergency readmission, relative risk for length of hospital stay; - c-statistics (a measure of discrimination) are not used in negative binomial models. Pseudo-R^2^ describes the variance explained by the model, calculated using Nagelkerke’s method for logistic regression models and McFadden’s for negative binomial models. * The model to estimate population attributable fraction included age and procedural risk as restricted cubic splines, all other models included age and procedural risk as simple continuous variables.

| **Disease combination** | **N (%)** | **Mean (SD) age** | **Emergency hospital readmission in 90 days** | |
| --- | --- | --- | --- | --- |
|  |  |  | **Crude rate**  **(%)** | **Adjusted odds ratio**  **(95% CI)** |
| **Elective** | | | | |
| RESP-DM | 372587 (2.2%) | 67.7 (12.7) | 59425 (16.0%) | 3.35 (3.32 – 3.39) |
| DM-CKD | 244744 (1.4%) | 72.0 (12.0) | 51672 (21.3%) | 4.51 (4.46 – 4.56) |
| RESP-Cancer | 220033 (1.3%) | 69.3 (12.5) | 47666 (21.9%) | 4.06 (4.01 – 4.10) |
| RESP-CKD | 177554 (1.0%) | 73.7 (12.0) | 38895 (22.2%) | 4.67 (4.61 – 4.73) |
| DM-Cancer | 172610 (1.0%) | 71.6 (10.3) | 34969 (20.5%) | 3.81 (3.76 – 3.85) |
| CCF-RESP | 166468 (1.0%) | 73.0 (12.0) | 39732 (24.3%) | 5.12 (5.06 – 5.18) |
| MI-DM | 151214 (0.9%) | 71.2 (10.6) | 29688 (19.8%) | 4.15 (4.09 – 4.21) |
| CCF-DM | 150445 (0.9%) | 72.4 (10.9) | 35554 (24.0%) | 5.15 (5.08 – 5.21) |
| PVD-DM | 142592 (0.8%) | 70.0 (11.2) | 31047 (22.0%) | 4.47 (4.41 – 4.53) |
| PVD-RESP | 140446 (0.8%) | 70.1 (12.5) | 30668 (22.2%) | 4.36 (4.30 – 4.42) |
| **Non-elective** | | | | |
| RESP-DM | 97179 (3.0%) | 69.4 (14.5) | 33021 (37.6%) | 3.09 (3.04 – 3.14) |
| DM-CKD | 94825 (2.9%) | 73.2 (13.1) | 35721 (42.8%) | 3.74 (3.68 – 3.79) |
| CCF-RESP | 86528 (2.7%) | 75.9 (12.4) | 30174 (42.0%) | 3.41 (3.35 – 3.47) |
| CCF-CKD | 79017 (2.4%) | 78.1 (12.1) | 28767 (44.7%) | 3.82 (3.75 – 3.89) |
| CCF-DM | 76460 (2.4%) | 74.1 (11.8) | 27574 (42.0%) | 3.60 (3.54 – 3.67) |
| RESP-CKD | 70928 (2.2%) | 75.9 (13.0) | 26108 (43.5%) | 3.64 (3.57 – 3.70) |
| PVD-DM | 68934 (2.1%) | 69.9 (12.8) | 25485 (41.1%) | 3.66 (3.60 – 3.72) |
| RESP-Cancer | 62043 (1.9%) | 72.0 (12.4) | 22919 (43.5%) | 2.96 (2.90 – 3.02) |
| PVD-RESP | 60240 (1.9%) | 71.8 (13.5) | 21438 (41.1%) | 3.39 (3.33 – 3.46) |
| PVD-CKD | 52762 (1.6%) | 74.1 (12.9) | 20278 (45.1%) | 4.04 (3.96 – 4.12) |

**Supplementary table 3. Hospital readmission for the ten commonest disease dyads, stratified by admission group.** Age reported as mean (standard deviation). CI; confidence interval. Odds ratio for hospital readmission adjusted for age, sex and procedure associated harm. Age included as a continuous variable without transformation. The reference category were patients with no long-term diseases. CCF; Cardiac failure, RESP; respiratory diseases, CKD; chronic kidney disease, DM; diabetes mellitus, MI; myocardial infarction, PVD; peripheral vascular disease, Rheum; rheumatological conditions.

| **Disease combination** | **N (%)** | **Mean (SD) age** | **Length of hospital stay** | |
| --- | --- | --- | --- | --- |
|  |  |  | **Median (IQR)** | **Adjusted relative risk**  **(95% CI)** |
| **Elective** | | | | |
| RESP-DM | 372587 (2.2%) | 67.7 (12.7) | 0 (0 to 1) | 1.74 (1.73 – 1.76) |
| DM-CKD | 244744 (1.4%) | 72.0 (12.0) | 0 (0 to 1) | 1.94 (1.93 – 1.96) |
| RESP-Cancer | 220033 (1.3%) | 69.3 (12.5) | 1 (0 to 4) | 2.42 (2.39 – 2.44) |
| RESP-CKD | 177554 (1.0%) | 73.7 (12.0) | 0 (0 to 2) | 2.03 (2.01 – 2.05) |
| DM-Cancer | 172610 (1.0%) | 71.6 (10.3) | 1 (0 to 3) | 2.36 (2.33 – 2.39) |
| CCF-RESP | 166468 (1.0%) | 73.0 (12.0) | 0 (0 to 2) | 2.21 (2.18 – 2.23) |
| MI-DM | 151214 (0.9%) | 71.2 (10.6) | 0 (0 to 2) | 1.73 (1.71 – 1.75) |
| CCF-DM | 150445 (0.9%) | 72.4 (10.9) | 0 (0 to 2) | 2.14 (2.11 – 2.16) |
| PVD-DM | 142592 (0.8%) | 70.0 (11.2) | 0 (0 to 3) | 2.38 (2.35 – 2.41) |
| PVD-RESP | 140446 (0.8%) | 70.1 (12.5) | 0 (0 to 3) | 2.29 (2.26 – 2.32) |
| **Non-elective** | | | | |
| RESP-DM | 97179 (3.0%) | 69.4 (14.5) | 7 (2 to 8) | 1.60 (1.58 – 1.61) |
| DM-CKD | 94825 (2.9%) | 73.2 (13.1) | 9 (2 to 22) | 1.84 (1.82 – 1.85) |
| CCF-RESP | 86528 (2.7%) | 75.9 (12.4) | 9 (3 to 23) | 1.61 (1.60-1.62) |
| CCF-CKD | 79017 (2.4%) | 78.1 (12.1) | 10 (3 to 24) | 1.64 (1.63 – 1.66) |
| CCF-DM | 76460 (2.4%) | 74.1 (11.8) | 8 (2 to 21) | 1.70 (1.68 – 1.71) |
| RESP-CKD | 70928 (2.2%) | 75.9 (13.0) | 9 (3 to 22) | 1.63 (1.62 – 1.65) |
| PVD-DM | 68934 (2.1%) | 69.9 (12.8) | 9 (3 to 24) | 2.16 (2.14 – 2.18) |
| RESP-Cancer | 62043 (1.9%) | 72.0 (12.4) | 8 (3 to 18) | 1.16 (1.15 – 1.17) |
| PVD-RESP | 60240 (1.9%) | 71.8 (13.5) | 9 (3 to 23) | 1.77 (1.75 – 1.79) |
| PVD-CKD | 52762 (1.6%) | 74.1 (12.9) | 10 (3 to 25) | 1.92 (1.90 – 1.94) |

**Supplementary table 4. Length of hospital stay for the ten commonest disease dyads, stratified by admission group.** Relative risk for hospital length of stay adjusted for age, sex and procedure associated harm, presented with 95% confidence intervals. IQR; interquartile range. Age is reported as mean (standard deviation). CI; confidence interval. Age included as a continuous variable without transformation. CI: Confidence interval. The reference category were patients with no long-term diseases. CCF; Cardiac failure, RESP; respiratory diseases, CKD; chronic kidney disease, DM; diabetes mellitus, MI; myocardial infarction, PVD; peripheral vascular disease, Rheum; rheumatological conditions.

| **Disease combination** | **Number (%)** | **Mean (SD) age** | **Number (%)** | | **Adjusted odds ratio of death (95%CI)** | |
| --- | --- | --- | --- | --- | --- | --- |
|  |  |  | **Death** | **Readmission** | **Age & Sex** | **Age & Sex & Procedure** |
| **Elective** | | | | | | |
| RESP-DM-CKD | 63,339 (0.4%) | 72.6 (11.1) | 2,766 (4.4%) | 16,104 (25.7%) | 11.04 (10.59 – 11.51) | 9.16 (8.77 – 9.56) |
| CCF-DM-CKD | 58,805 (0.3%) | 73.4 (10.9) | 3,592 (6.1%) | 17,140 (29.7%) | 14.79 (14.25 – 15.36) | 11.93 (11.47 – 12.41) |
| CCF-RESP-DM | 53,701 (0.3%) | 72.6 (10.3) | 2,859 (5.3%) | 14,760 (28%) | 13.78 (13.22 – 14.35) | 10.95 (10.39 – 11.43) |
| CCF-RESP-CKD | 49,385 (0.3%) | 75.6 (10.8) | 3,521 (7.1%) | 14,575 (30.2%) | 15.50 (14.91 – 16.10) | 12.40 (11.92 – 12.91) |
| PVD-DM-CKD | 44,616 (0.3%) | 71.1 (11.6) | 2,495 (5.6%) | 13,000 (29.7%) | 14.78 (14.14 – 15.44) | 11.42 (10.91 – 11.95) |
| CCF-MI-DM | 43,162 (0.3%) | 72.3 (10.3) | 2,192 (5.1%) | 11,749 (27.7%) | 12.74 (12.16 – 13.34) | 10.12 (9.64 – 10.63) |
| MI-RESP-DM | 41,946 (0.2%) | 71.5 (10.2) | 1,502 (3.6%) | 10,621 (25.6%) | 9.36 (8.87 – 9.89) | 7.75 (7.33 – 8.21) |
| RESP-DM-Cancer | 40,282 (0.2%) | 71.9 (9.9) | 2,441 (6.1%) | 9,675 (24.3%) | 16.26 (15.56 – 17.00) | 10.91 (10.41 – 11.44) |
| MI-DM-CKD | 38,831 (0.2%) | 73.5 (10.4) | 1,912 (4.9%) | 10,936 (28.6%) | 11.38 (10.38 – 11.95) | 9.43 (8.96 – 9.93) |
| CCF-MI-RESP | 38,802 (0.2%) | 73.9 (10.3) | 2,146 (5.5%) | 11,143 (29.2%) | 12.72 (12.14 – 13.33) | 10.11(9.63 – 10.62) |
| **Non-Elective** | | | | | | |
| CCF-DM-CKD | 34,616 (1.1%) | 75.1 (11.4) | 8,763 (25.3%) | 13,983 (48.3%) | 6.63(6.44 – 6.82) | 5.68 (5.52 – 5.85) |
| CCF-RESP-CKD | 30,224 (0.9%) | 77.7 (11.3) | 9,125 (30.2%) | 11,797 (48.6%) | 7.40 (7.19 – 7.62) | 6.33 (6.14 – 6.53) |
| CCF-RESP-DM | 27,615 (0.9%) | 74.1 (11.1) | 6,662 (24.1%) | 10,941 (46.9%) | 6.69 (6.49 – 6.90) | 5.39 (5.21 – 5.57) |
| RESP-DM-CKD | 27,196 (0.8%) | 73.8 (12.1) | 6,200 (22.8%) | 11,096 (47.5%) | 6.16 (5.97 – 6.36) | 4.99 (4.82 – 5.16) |
| PVD-DM-CKD | 26,734 (0.8%) | 71.6 (12.4) | 5,561 (20.8%) | 11,321 (48.7%) | 5.97 (5.77 – 6.7) | 5.31 (5.13 – 5.50) |
| CCF-PVD-CKD | 21,679 (0.7%) | 75.6 (11.7) | 6,212 (28.7%) | 8,733 (49.6%) | 7.51 (7.26 – 7.76) | 6.67 (6.44 – 6.91) |
| CCF-MI-DM | 21,159 (0.7%) | 73.8 (11.1) | 4,405 (20.8%) | 8,662 (46.8%) | 5.39 (5.20 – 5.59) | 4.87 (4.69 – 5.07) |
| CCF-PVD-DM | 20,930 (0.6%) | 72.9 (11.1) | 4,873 (23.3%) | 8,643 (48.6%) | 6.62 (6.39 – 6.86) | 5.89 (5.67 – 6.11) |
| CCF-MI-CKD | 20,546 (0.6%) | 77.7 (10.9) | 5,589 (27.2%) | 8,444 (49.5%) | 6.15 (5.94 – 6.37) | 5.71 (5.51 – 5.93) |
| CCF-MI-RESP | 19,867 (0.6%) | 76 (11) | 4,793 (24.1%) | 8,145 (48.1%) | 5.84 (5.63 – 6.05) | 5.07 (4.88 – 5.27) |

**Supplementary table 5. Ten commonest triads of disease, stratified by admission group.** Death; Death within 90 days of surgical procedure. Readmission; Emergency hospital readmission within 90 days of surgical procedure. SD; standard deviation. Odds ratio adjusted compared to patients with no diseases using logistic regression models. Procedure: model included procedure associated harm at 90 days. Age included as a continuous variable without transformation. CCF; Cardiac failure, RESP; respiratory diseases, CKD; chronic kidney disease, DM; diabetes mellitus, MI; myocardial infarction, PVD; peripheral vascular disease, Rheum; rheumatological conditions.

| **Disease combination** | **Number (%)** | **Mean (SD) age** | **Number (%)** | | **Adjusted odds ratio of death (95%CI)** | |
| --- | --- | --- | --- | --- | --- | --- |
|  |  |  | **Death** | **Readmission** | **Age & Sex** | **Age & Sex & Procedure** |
| **Elective** | | | | | | |
| CCF-RESP-DM-CKD | 22,146 (0.1%) | 73.9 (10.1) | 1,540 (7%) | 7,182 (33.1%) | 16.77 (15.87 – 7.71) | 13.44 (12.69 – 14.23) |
| CCF-MI-DM-CKD | 18,465 (0.1%) | 73.4 (10.3) | 1,244 (6.7%) | 6,091 (33.7%) | 16.00 (15.06 – 17.00) | 13.09 (12.28 – 13.94) |
| CCF-PVD-DM-CKD | 16,154 (0.1%) | 72.1 (10.9) | 1,356 (8.4%) | 5,573 (35.6%) | 21.83 (20.59 – 23.16) | 16.83 (15.83 – 17.89) |
| CCF-MI-RESP-DM | 15,599 (0.1%) | 72.7 (9.8) | 893 (5.7%) | 5,016 (32.8%) | 14.39 (13.41 – 15.43) | 11.49 (10.67 – 12.37) |
| CCF-MI-RESP-CKD | 14,024 (0.1%) | 75.8 (9.7) | 1,070 (7.6%) | 4,759 (34.7%) | 15.98 (14.97 – 17.06) | 13.12 (12.26 – 14.05) |
| PVD-RESP-DM-CKD | 13,174 (0.1%) | 72 (10.8) | 921 (7%) | 4,384 (34.1%) | 18.06 (16.84 – 19.37) | 14.07 (13.09 – 15.13) |
| MI-RESP-DM-CKD | 12,780 (0.1%) | 73.7 (9.7) | 747 (5.8%) | 4,237 (33.7%) | 13.78 (12.76 – 14.87) | 11.39 (10.51 – 12.34) |
| CCF-PVD-RESP-CKD | 12,770 (0.1%) | 74.3 (10.4) | 1,206 (9.4%) | 4,402 (35.6%) | 21.93 (20.60 – 23.34) | 16.55 (15.50 – 17.66) |
| CCF-MI-PVD-DM | 12,555 (0.1%) | 71.6 (10.1) | 874 (7%) | 4,097 (33.5%) | 18.42 (17.15 – 19.79) | 14.28 (13.24 – 15.39) |
| CCF-PVD-RESP-DM | 12,028 (0.1%) | 72.1 (10) | 923 (7.7%) | 4,064 (34.7%) | 20.32 (18.95 – 21.79) | 15.54 (14.45 – 16.72) |
| **Non-Elective** | | | | | | |
| CCF-RESP-DM-CKD | 13,330 (0.4%) | 75.1 (10.7) | 3,617 (27.1%) | 5,677 (51.6%) | 7.40 (7.10 – 7.71) | 6.25 (5.98 – 6.53) |
| CCF-PVD-DM-CKD | 11,820 (0.4%) | 73.2 (11.2) | 3,033 (25.7%) | 5,219 (52.8%) | 7.41 (7.09 – 7.75) | 6.72 (6.41 – 7.04) |
| CCF-MI-DM-CKD | 10,875 (0.3%) | 74.8 (10.8) | 2,682 (24.7%) | 4,892 (52.9%) | 6.39 (6.10 – 6.70) | 5.87 (5.58 – 6.17) |
| CCF-PVD-RESP-CKD | 8,886 (0.3%) | 75.8 (10.9) | 2,688 (30.2%) | 3,746 (52.6%) | 8.16 (7.77 – 8.57) | 7.17 (6.81 – 7.55) |
| CCF-MI-RESP-CKD | 8,531 (0.3%) | 77.3 (10.4) | 2,404 (28.2%) | 3,768 (53.6%) | 6.70 (6.37 – 7.05) | 6.07 (5.75 – 6.40) |
| PVD-RESP-DM-CKD | 8,303 (0.3%) | 72.4 (11.5) | 2,001 (24.1%) | 3,749 (53.1%) | 7.10 (6.73 – 7.49) | 6.21 (5.87 – 6.58) |
| CCF-MI-RESP-DM | 8,221 (0.3%) | 73.7 (10.5) | 1,838 (22.4%) | 3,737 (52.6%) | 6.03 (5.71 – 6.37) | 5.25 (4.95 – 5.57) |
| CCF-PVD-RESP-DM | 8,126 (0.3%) | 73 (10.6) | 2,012 (24.8%) | 3,564 (52.1%) | 7.22 (6.84 – 7.62) | 6.27 (5.92 – 6.64) |
| CCF-MI-PVD-DM | 7,809 (0.2%) | 72.2 (10.7) | 1,719 (22%) | 3,566 (52.7%) | 6.31 (5.96 – 6.68) | 5.85 (5.51 – 6.21) |
| CCF-MI-PVD-CKD | 7,636 (0.2%) | 75.3 (10.8) | 2,126 (27.8%) | 3,405 (54.1%) | 7.25 (6.87 – 7.65) | 6.72 (6.35 – 7.11) |

**Supplementary table 6. Ten commonest quads of disease, stratified by admission group.** Death; Death within 90 days of surgical procedure. Readmission; Emergency hospital readmission within 90 days of surgical procedure. SD; standard deviation. Odds ratio compared to patients with no diseases with logistic regression models. Procedure: model included procedure associated harm at 90 days. Age included as a continuous variable without transformation. CCF; Cardiac failure, RESP; respiratory diseases, CKD; chronic kidney disease, DM; diabetes mellitus, MI; myocardial infarction, PVD; peripheral vascular disease, Rheum; rheumatological conditions.

| **Disease combination** | **Number (%)** | **Mean (SD) age** | **Number (%)** | | **Adjusted odds ratio (95%CI)** | |
| --- | --- | --- | --- | --- | --- | --- |
|  |  |  | **Death** | **Readmission** | **Age & Sex** | **Age & Sex & Procedure** |
| **Elective** | | | | | | |
| CCF-Liver | 15,014 (0.1) | 66.7 (13.6) | 1,339 (8.9%) | 4,311 (30.3%) | 31.83 (29.99 – 33.78) | 20.06 (18.80 – 21.40) |
| Hemi-Para-Cancer | 8,585 (0.1) | 68.6 (13.7) | 748 (8.7%) | 2,409 (28.7%) | 26.45 (24.45 – 28.61) | 15.97 (14.67 – 17.38) |
| Liver-Cancer | 30,410 (0.2) | 65.1 (12.1) | 2,488 (8.2%) | 7,862 (26.5%) | 34.00 (32.53 – 35.55) | 17.24 (16.44 – 18.09) |
| CCF-Cancer | 56,356 (0.3) | 75.6 (10.4) | 4,596 (8.2%) | 14,358 (26.1%) | 17.58 (16.98 – 18.21) | 12.23 (11.79 – 12.69) |
| Dementia-Cancer | 14,833 (0.1) | 80 (8.7) | 1,193 (8%) | 3,700 (25.3%) | 13.04 (12.25 – 13.88) | 10.77 (10.09 – 11.50) |
| CCF-Dementia | 14,988 (0.1) | 81.3 (9) | 1,199 (8%) | 4,246 (28.9%) | 12.54 (11.79 – 13.35) | 11.05 (10.35 – 11.78) |
| PVD-Dementia | 10,464 (0.1) | 78.7 (9.3) | 778 (7.4%) | 2,870 (28%) | 13.00 (12.05 – 14.03) | 10.61 (9.81 – 11.48) |
| CVA-Cancer | 36,539 (0.2) | 74.9 (10.8) | 2,685 (7.3%) | 9,150 (25.6%) | 16.11 (15.43 – 16.83) | 10.50 (10.03 – 11.00) |
| PVD-Liver | 13,585 (0.1) | 64.4 (13.8) | 939 (6.9%) | 3,701 (28.3%) | 27.13 (25.30 – 29.08) | 16.08 (14.93 – 17.32) |
| Dementia-CKD | 21,354 (0.1) | 81.5 (8.9) | 1,393 (6.5%) | 5,534 (26.3%) | 10.05 (9.48 – 10.65) | 9.09 (8.57 – 9.65) |
| **Non-Elective** | | | | | | |
| CCF-Cancer | 27,875 (0.9) | 77.9 (10.5) | 10,579 (38%) | 9,741 (43.8%) | 10.72 (10.41 – 11.03) | 6.72 (6.52 – 6.93) |
| Liver-Cancer | 11,296 (0.3) | 66.4 (13) | 4,262 (37.7%) | 4,236 (45.2%) | 21.43 (20.54 – 22.36) | 9.59 (9.15 – 10.05) |
| RESP-Cancer | 62,043 (1.9) | 72 (12.4) | 22,953 (37%) | 22,919 (43.5%) | 15.19 (14.87 – 15.51) | 7.56 (7.39 – 7.74) |
| Dementia-Cancer | 13,517 (0.4) | 83.2 (8.1) | 4,913 (36.3%) | 4,199 (36.7%) | 7.07 (6.80 – 7.35) | 6.25 (6.00 – 6.52) |
| CKD-Cancer | 36,919 (1.1) | 77.5 (10.8) | 13,053 (35.4%) | 13,908 (45.1%) | 9.69 (9.44 – 9.94) | 6.21 (6.04 – 6.38) |
| CCF-Liver | 11,548 (0.4) | 67.2 (15) | 4,050 (35.1%) | 3,908 (46.6%) | 17.05 (16.33 – 17.80) | 10.51 (10.03 – 11.01) |
| CVA-Cancer | 18,499 (0.6) | 77.1 (11.2) | 6,382 (34.5%) | 6,293 (40.7%) | 9.34 (9.02 – 9.67) | 5.93 (5.72 – 6.16) |
| PVD-Cancer | 23,515 (0.7) | 74.1 (11.5) | 7,900 (33.6%) | 8,702 (43.9%) | 10.63 (10.30 – 10.96) | 6.60 (6.38 – 6.83) |
| CCF-Dementia | 27,801 (0.9) | 84.9 (8.2) | 9,300 (33.5%) | 8,924 (39.7%) | 5.91 (5.74 – 6.09) | 6.17 (5.98 – 6.36) |
| Hemi-Para-Cancer | 4,925 (0.2) | 70.8 (13.4) | 1,612 (32.7%) | 1,780 (42.1%) | 12.11 (11.35 – 12.91) | 7.35 (6.86 – 7.88) |

**Supplementary table 7. Ten disease dyads associated with the greatest rate of death, stratified by admission group.** Death; Death within 90 days of surgical procedure. Readmission; Emergency hospital readmission within 90 days of surgical procedure. SD; standard deviation. Odds ratio adjusted compared to patients with no diseases using logistic regression models. Procedure: model included procedure associated harm at 90 days. Age included as a continuous variable without transformation. CCF; Cardiac failure, RESP; respiratory diseases, CKD; chronic kidney disease, DM; diabetes mellitus, MI; myocardial infarction, PVD; peripheral vascular disease, Rheum; rheumatological conditions.

|  | | **Age-standardised ratio of death compared to base disease and at least one other disease** | | | | | | | | | | | |
| --- | --- | --- | --- | --- | --- | --- | --- | --- | --- | --- | --- | --- | --- |
|  |  | **CCF** | **CKD** | **Respiratory** | **Stroke** | **Cancer** | **Diabetes** | **Dementia** | **Paraplegia** | **Liver** | **MI** | **PVD** | **Rheum** |
| **Base disease (% dead in 90 days)** | CCF (3.3%) |  | 1.2 (1.1-1.4) | 1 (0.9-1.1) | 1.4 (1.1-2.1) | 2.6 (2.1-3.5) | 1 (0.8-1.4) | 1.5 (0.7-6.1) | 1.4 (0.9-2.6) | 2.4 (1.9-3) | 1 (0.7-2.2) | 1.3 (1.1-1.4) | 1.1 (0.7-2.1) |
|  | CKD (2.4%) | 1.7 (1.6-2) |  | 1 (0.9-1.1) | 1.7 (1.3-2.6) | 2.6 (2.1-3.3) | 0.9 (0.8-1.1) | 2.6 (1.3-8.3) | 1.5 (1-3.2) | 2.1 (1.7-2.6) | 1.4 (1-4.8) | 1.4 (1.3-1.7) | 0.8 (0.6-1.2) |
|  | Respiratory (1.8%) | 1.9 (1.7-2.2) | 1.4 (1.2-1.6) |  | 1.4 (1.1-1.9) | 2.4 (2.3-2.6) | 0.7 (0.6-0.8) | 1.3 (0.9-4) | 1.2 (0.9-1.6) | 1.8 (1.5-2.1) | 1.1 (0.8-2.7) | 1.4 (1.2-1.6) | 0.6 (0.5-0.7) |
|  | Stroke (2.7%) | 1.8 (1.4-2.5) | 1.5 (1.2-2.1) | 0.9 (0.8-1.1) |  | 3.2 (2.4-4.1) | 0.9 (0.7-1.8) | 1 (0.8-5.5) | 0.7 (0.6-0.9) | 2.7 (1.8-4.1) | 0.9 (0.7-3.8) | 1.1 (1-1.5) | 0.7 (0.5-2.4) |
|  | Cancer (4.6%) | 1.9 (1.4-2.6) | 1.3 (1.1-1.8) | 0.9 (0.9-1) | 1.8 (1.3-2.6) |  | 0.9 (0.7-1.2) | 1.1 (0.7-5) | 1.7 (1.2-2.4) | 1.7 (1.4-2.1) | 1.2 (0.7-4.3) | 1.3 (1-1.5) | 0.6 (0.5-1.9) |
|  | DM (1.5%) | 2.3 (1.9-3.2) | 1.5 (1.3-1.8) | 0.8 (0.8-0.9) | 1.7 (1.2-3.5) | 2.8 (2.3-3.6) |  | 1.8 (1-8.2) | 1.5 (1.1-2.9) | 2.1 (1.7-2.6) | 1.2 (0.9-3.5) | 1.9 (1.5-2.4) | 0.8 (0.5-2.1) |
|  | Dementia (3.4%) | 1.5 (1-3.7) | 1.8 (1.2-3.6) | 0.7 (0.7-1.2) | 0.8 (0.8-2.9) | 1.5 (1.3-4) | 0.8 (0.6-2.2) |  | 0.8 (0.6-2.2) | 2.5 (1.5-2.9) | 2.4 (0.7-5.5) | 1.4 (0.9-4.4) | 0.6 (0.3-3.5) |
|  | Hemi-Para (2.2%) | 2.2 (1.5-3.6) | 1.6 (1.2-3.1) | 0.9 (0.8-1.1) | 0.9 (0.8-1.1) | 3.5 (2.8-4.4) | 1 (0.8-1.7) | 1.3 (0.8-5) |  | 2.1 (0.9-4.4) | 1.2 (0.8-6.6) | 1.5 (1-2.4) | 0.6 (0.3-2.8) |
|  | Liver (3.8%) | 2.1 (1.7-2.6) | 1.3 (1.1-1.6) | 0.8 (0.8-0.9) | 1.9 (1.2-3.1) | 2.1 (1.8-2.5) | 0.8 (0.7-1) | 2.2 (1-4.2) | 1.3 (0.5-2.8) |  | 1.4 (0.7-4.3) | 1.6 (1.2-2) | 0.6 (0.4-1.5) |
|  | MI (1.9%) | 1.8 (1.5-2.3) | 1.8 (1.6-3.4) | 1 (0.9-1.4) | 1.2 (1.1-3.3) | 2.9 (2-5.8) | 0.9 (0.9-1.5) | 4.3 (1-9.2) | 1.4 (1-4.9) | 2.7 (1.7-5) |  | 1.3 (1.2-2.6) | 0.7 (0.6-3.7) |
|  | PVD (2.5%) | 1.7 (1.5-1.9) | 1.3 (1.2-1.6) | 1 (0.9-1.1) | 1.2 (1-1.7) | 2.4 (2-2.8) | 1.1 (0.9-1.4) | 1.9 (0.9-9.9) | 1.3 (0.8-2.4) | 2.4 (1.8-3.1) | 1 (0.8-3.5) |  | 0.7 (0.6-1.1) |
|  | Rheum (1%) | 3.4 (2.3-6.1) | 1.7 (1.4-2.5) | 0.9 (0.9-1) | 1.9 (1.2-5.9) | 2.8 (2.3-7.2) | 1.1 (0.8-2.6) | 2 (0.8-16.4) | 1.4 (0.6-6) | 2.3 (1.4-4.8) | 1.3 (1-10.5) | 1.7 (1.4-2.2) |  |

|  | **Ratio > 2** |  |  | **Ratio 1-2** |  |  | **Ratio < 1** |
| --- | --- | --- | --- | --- | --- | --- | --- |

**Supplementary table 8**. **The age-standardised relative risk of death within 90 days after elective surgery associated with each additional disease, compared to the rate associated with the base disease and more than one other disease.** The number in the base disease column is the age-standardised rate of death within 90 days associated with each disease with at least one other disease. CCF; Cardiac failure, CKD; chronic kidney disease, DM; diabetes mellitus, MI; myocardial infarction, PVD; peripheral vascular disease, Rheum; rheumatological conditions.

|  | | **Age-standardised ratio of death compared to base disease and at least one other disease** | | | | | | | | | | | |
| --- | --- | --- | --- | --- | --- | --- | --- | --- | --- | --- | --- | --- | --- |
|  |  | **CCF** | **CKD** | **Respiratory** | **Stroke** | **Cancer** | **DM** | **Dementia** | **Hemi-Para** | **Liver** | **MI** | **PVD** | **Rheum** |
| **Base disease (% dead in 90 days)** | CCF (15.4%) | - | 1 (0.9-1.1) | 1 (1-1.1) | 1.2 (1-1.5) | 2.3 (1.8-2.7) | 0.9 (0.8-1.1) | 1.7 (1-2.7) | 0.9 (0.6-1.5) | 1.9 (1.7-2) | 1.1 (0.7-1.6) | 1 (0.9-1.2) | 1.1 (0.8-1.5) |
|  | CKD (11.6%) | 1.3 (1.2-1.4) | - | 1.1 (1-1.1) | 1.3 (1.1-1.7) | 2.4 (2-2.9) | 0.9 (0.8-1) | 1.4 (0.9-2.7) | 1.1 (0.8-1.7) | 1.6 (1.5-1.8) | 1.3 (0.9-2.1) | 1.1 (1-1.3) | 0.9 (0.7-1.1) |
|  | Respiratory (11.6%) | 1.4 (1.2-1.5) | 1.1 (1-1.2) | - | 1.3 (1.1-1.6) | 2.5 (2.4-2.7) | 0.7 (0.7-0.7) | 1.3 (0.9-2.2) | 0.8 (0.7-1) | 1.3 (1.3-1.4) | 0.8 (0.7-1.2) | 0.9 (0.9-1) | 0.7 (0.6-0.8) |
|  | Stroke (13.7%) | 1.3 (1.1-1.6) | 1.1 (1-1.4) | 1.1 (1-1.2) | - | 2.4 (2.1-2.8) | 1 (0.8-1.3) | 1 (0.7-2.1) | 0.6 (0.6-0.7) | 1.5 (1.2-1.9) | 0.8 (0.7-1.6) | 1.1 (0.9-1.4) | 0.8 (0.6-1.4) |
|  | Cancer (28.4%) | 1.2 (0.9-1.5) | 1 (0.8-1.2) | 1 (1-1.1) | 1.2 (1-1.4) | - | 0.9 (0.8-1.1) | 0.9 (0.5-1.7) | 0.9 (0.7-1.2) | 1.1 (1-1.3) | 0.8 (0.5-1.2) | 1 (0.9-1.2) | 0.9 (0.6-1.4) |
|  | DM (8.6%) | 1.7 (1.4-2.1) | 1.2 (1.1-1.3) | 0.9 (0.9-1) | 1.5 (1.2-2.2) | 3.1 (2.5-3.7) | - | 1.8 (1.2-3.3) | 1 (0.8-1.6) | 1.7 (1.6-1.9) | 0.9 (0.8-2) | 1.1 (0.9-1.2) | 0.8 (0.7-1.2) |
|  | Dementia (16.3%) | 1.6 (1-2.2) | 1 (0.8-1.6) | 0.9 (0.8-1.2) | 0.8 (0.6-1.5) | 1.5 (1.1-2.5) | 0.9 (0.8-1.4) | - | 0.6 (0.4-1.2) | 1.3 (1.3-1.6) | 0.7 (0.5-1.6) | 0.9 (0.7-1.8) | 0.5 (0.3-2.1) |
|  | Paraplegia (9.3%) | 1.5 (1.1-2.4) | 1.4 (1.1-2) | 1 (0.9-1.1) | 0.9 (0.9-1.1) | 2.9 (2.4-3.5) | 1 (0.8-1.3) | 1 (0.7-2.4) | - | 1.4 (1-2.4) | 1.2 (0.7-2.4) | 1.2 (1-1.6) | 0.9 (0.6-2.1) |
|  | Liver (17.4%) | 1.7 (1.5-1.8) | 1.1 (1-1.2) | 0.9 (0.9-0.9) | 1.2 (0.9-1.6) | 1.9 (1.6-2.1) | 0.9 (0.8-0.9) | 1.2 (1-1.9) | 0.7 (0.5-1.4) | - | 0.8 (0.7-1.3) | 1 (0.9-1.1) | 0.9 (0.6-1.3) |
|  | MI (8.8%) | 1.9 (1.3-2.3) | 1.7 (1.4-2.1) | 1 (1-1.2) | 1.3 (1.1-2.1) | 2.7 (2-3.1) | 0.9 (0.9-1.5) | 1.3 (1-2.8) | 1.2 (0.8-2.2) | 1.6 (1.6-2) | - | 1.3 (1-1.7) | 0.7 (0.7-1.7) |
|  | PVD (10.7%) | 1.5 (1.3-1.7) | 1.2 (1.1-1.4) | 1 (1-1.1) | 1.4 (1.1-1.9) | 2.7 (2.4-3.1) | 0.9 (0.8-1) | 1.4 (1-3.3) | 1 (0.8-1.5) | 1.6 (1.4-1.8) | 1.1 (0.7-1.8) | - | 0.9 (0.7-1.3) |
|  | Rheum (8.3%) | 2 (1.5-2.7) | 1.2 (1-1.5) | 1 (0.9-1) | 1.4 (1-2.3) | 2.9 (2.1-4.4) | 0.8 (0.7-1.2) | 1 (0.6-4.7) | 1 (0.7-2.4) | 1.8 (1.4-2.6) | 0.8 (0.6-2.2) | 1.2 (1-1.5) | - |

|  | **Ratio > 2** |  |  | **Ratio 1-2** |  |  | **Ratio < 1** |
| --- | --- | --- | --- | --- | --- | --- | --- |

**Supplementary table 9**. **The age-standardised relative risk of death within 90 days after non-elective surgery associated with each additional disease, compared to the rate associated with the base disease and more than one other disease.** The number in the base disease column is the age-standardised rate of death within 90 days associated with each disease with at least one other disease. CCF; Cardiac failure, CKD; chronic kidney disease, DM; diabetes mellitus, MI; myocardial infarction, PVD; peripheral vascular disease, Rheum; rheumatological conditions.

|  | **All patients** | | **No multi-morbidity** | | **Multi-morbidity** | |
| --- | --- | --- | --- | --- | --- | --- |
|  | n | High-risk procedure (%) | n | High-risk procedure (%) | n | High-risk procedure (%) |
| Non-Elective | 3,246,851 | 1,438,570 (44.3) | 2,572,661 | 956,945 (37.2) | 674,190 | 481,625 (71.4) |
| Elective | 16,946,808 | 312,336 (1.8) | 15,04,3949 | 204,251 (1.4) | 1,902,859 | 1,080,85 (5.7) |
| Any urgency | 20,193,659 | 1,750,906 (8.7) | 17,616,610 | 1,161,196 (6.6) | 2,577,049 | 589,710 (22.9) |

**Supplementary table 10.** **Proportion of patients undergoing a high-risk procedure, stratified by admission category.** High-risk procedures were defined as those with an aggregate 90-day risk of death in greater than or equal to 5%.

|  |  | **Adjusted Odds Ratio (95% Confidence interval) for 90-day death** | |
| --- | --- | --- | --- |
| **Disease combination** | **Setting** | **Age & sex & procedure** | **Age & sex & procedure**  **Random intercept: provider code** |
| **Commonest combinations** | | | |
| RESP-DM | Elective | 5.65 (5.50 – 5.81) | 5.31 (5.11 – 5.18) |
| DM-CKD | Elective | 7.16 (6.96 – 7.37) | 6.82 (6.41 – 6.97) |
| RESP-DM | Non-elective | 3.82 (3.73 – 3.91) | 3.67 (3.40 – 3.93) |
| DM-CKD | Non-elective | 4.22 (4.13 – 4.31) | 4.22 (4.08 – 4.37) |
| **Highest risk combinations** | | | |
| CCF-Liver | Elective | 20.06 (18.80 – 21.40) | 18.27 (16.50 – 20.02) |
| HemiPara – Cancer | Elective | 15.97 (14.67 – 17.38) | 14.78 (13.30 – 16.44) |
| CCF-Cancer | Emergency | 6.72 (6.52 – 6.93) | 6.79 (6.53 – 7.05) |
| Liver-Cancer | Emergency | 9.59 (9.15 – 10.05) | 9.65 (8.95 – 10.40) |

**Supplementary table 11. Multi-level models in selected disease dyads.** Adjusted odds ratios from logistic regression models. The random intercept model included fixed effects of age, sex and procedure associated harm, and a random intercept for provider codes. CCF; Cardiac failure, RESP; respiratory diseases, CKD; chronic kidney disease, DM; diabetes mellitus, MI; myocardial infarction, PVD; peripheral vascular disease, Rheum; rheumatological conditions, Hemi-Para; hemi-paraplegia.

**
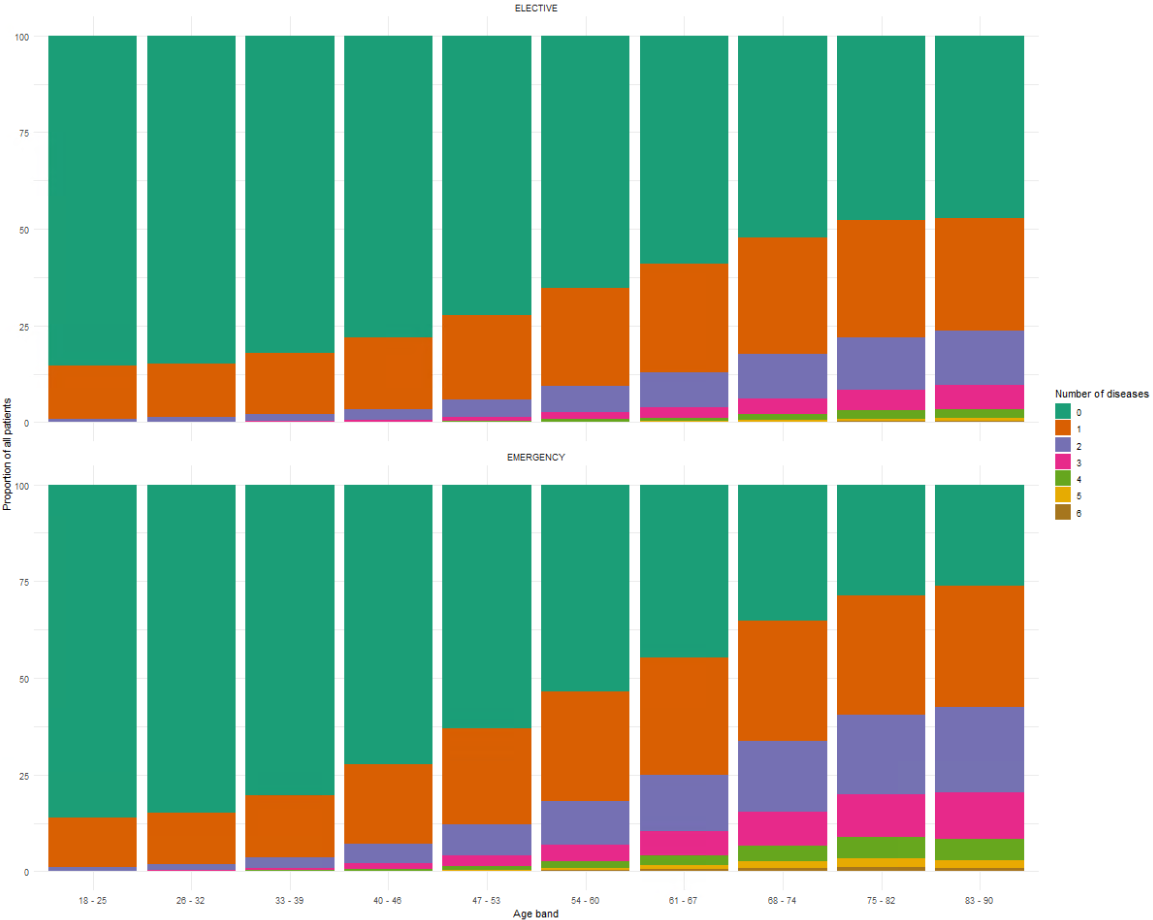
**

|  | **18-25** | **26-32** | **33-39** | **40-46** | **47-53** | **54-60** | **61-67** | **68-74** | **75-82** | **82-≥90** |
| --- | --- | --- | --- | --- | --- | --- | --- | --- | --- | --- |
| Elective | 1010170 | 1113507 | 1222950 | 1645922 | 1878188 | 1894230 | 2326478 | 2291906 | 2287849 | 1275608 |
| Non-elective | 350192 | 300301 | 263371 | 268264 | 277044 | 272105 | 314787 | 319704 | 409284 | 471799 |

**Supplementary figure 1. The proportion of patients with differing number of diseases, stratified to 10 bands of age and presented divided by admission group.** Numbers in each group in the table below.

**
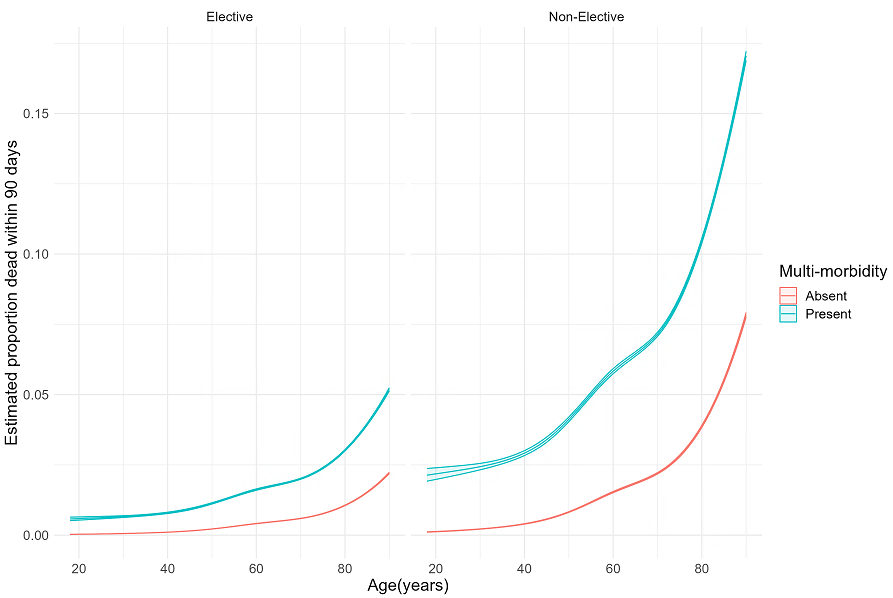
**

**Supplementary figure 2. Estimated proportion of patients dying within 90 days of surgery, modelled with an interaction term between the presence of multi-morbidity and age.** Age was modelled as a restricted cubic spline with five knots, the model also included sex, setting and procedure associated harm which was modelled with a restricted cubic spline with three knots. Events: 359,494; pseudo-R2 0.341; C0.920.


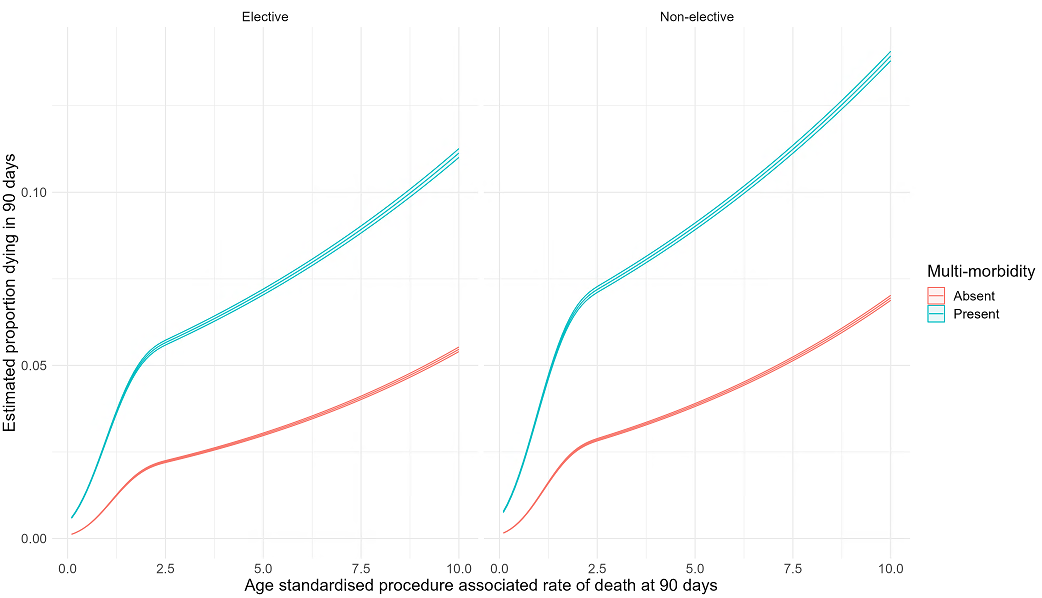


**Supplementary figure 3. Estimated proportion of patients dying within 90 days of surgery, modelled with an interaction term between the presence of multi-morbidity and procedure associated rate of death.** Age-standardised procedure associated harm which was modelled with a restricted cubic spline with three knots and is a percentage estimated to die in 90 days, the model also included sex, setting and age modelled as a restricted cubic spline with five knots. Events: 359,494; pseudo-R2 0.370; C0.932.

**Appendix A:** Disease combinations for two to four diseases, with a crude prevalence exceeding 1 in 2000 and with a crude rate of death of more than 1 in 20 (5%).

| **Combination** | **Prevalence per 1000 spells** | **Rate of death in 90 days (%)** |
| --- | --- | --- |
| CCF-PVD | 5.05 | 6.34 |
| CCF-CVA | 2.78 | 6.4 |
| CCF-Dementia | 0.88 | 8 |
| CCF-Liver | 0.89 | 8.92 |
| CCF-Hemi-Para | 0.53 | 6.13 |
| CCF-CKD | 7.78 | 6.22 |
| CCF-Cancer | 3.33 | 8.16 |
| MI-Dementia | 0.68 | 5.73 |
| MI-Liver | 0.55 | 5.5 |
| MI-Cancer | 3.43 | 5.39 |
| PVD-CVA | 2.89 | 5.08 |
| PVD-Dementia | 0.62 | 7.44 |
| PVD-Liver | 0.8 | 6.91 |
| PVD-CKD | 5.98 | 5.4 |
| PVD-Cancer | 4 | 6.39 |
| CVA-Dementia | 1.5 | 5.75 |
| CVA-CKD | 3.2 | 5.41 |
| CVA-Cancer | 2.16 | 7.35 |
| Dementia-RESP | 1.53 | 5.19 |
| Dementia-CKD | 1.26 | 6.52 |
| Dementia-Cancer | 0.88 | 8.04 |
| RESP-Cancer | 12.98 | 5.71 |
| Liver-CKD | 1.21 | 5.95 |
| Liver-Cancer | 1.79 | 8.18 |
| Hemi-Para-CKD | 0.65 | 5.17 |
| Hemi-Para-Cancer | 0.51 | 8.71 |
| CKD-Cancer | 5.66 | 6.51 |
| CCF-MI-PVD | 1.64 | 6.66 |
| CCF-MI-CVA | 0.8 | 6.79 |
| CCF-MI-RESP | 2.29 | 5.53 |
| CCF-MI-DM | 2.55 | 5.08 |
| CCF-MI-CKD | 2.13 | 6.87 |
| CCF-MI-Cancer | 0.77 | 7.8 |
| CCF-PVD-CVA | 0.73 | 8.34 |
| CCF-PVD-RESP | 1.91 | 7.31 |
| CCF-PVD-DM | 1.89 | 6.81 |
| CCF-PVD-CKD | 1.9 | 8.41 |
| CCF-PVD-Cancer | 0.59 | 9.34 |
| CCF-CVA-RESP | 1 | 7.25 |
| CCF-CVA-DM | 1 | 6.53 |
| CCF-CVA-CKD | 0.98 | 8.11 |
| CCF-RESP-Rheum | 0.75 | 5.24 |
| CCF-RESP-DM | 3.17 | 5.32 |
| CCF-RESP-CKD | 2.91 | 7.13 |
| CCF-RESP-Cancer | 1.22 | 9.1 |
| CCF-Rheum-CKD | 0.56 | 6.13 |
| CCF-DM-CKD | 3.47 | 6.11 |
| CCF-DM-Cancer | 0.99 | 7.97 |
| CCF-CKD-Cancer | 1.01 | 9.48 |
| MI-PVD-CVA | 0.63 | 6.11 |
| MI-PVD-RESP | 1.6 | 5.27 |
| MI-PVD-CKD | 1.38 | 6.72 |
| MI-PVD-Cancer | 0.66 | 6.57 |
| MI-CVA-RESP | 0.74 | 5.31 |
| MI-CVA-CKD | 0.64 | 6.71 |
| MI-RESP-CKD | 1.65 | 5.8 |
| MI-RESP-Cancer | 1.01 | 6.89 |
| MI-DM-Cancer | 0.98 | 5.73 |
| MI-CKD-Cancer | 0.74 | 7.41 |
| PVD-CVA-RESP | 0.89 | 6.15 |
| PVD-CVA-DM | 0.97 | 5.47 |
| PVD-CVA-CKD | 0.82 | 7.49 |
| PVD-RESP-CKD | 1.84 | 6.77 |
| PVD-RESP-Cancer | 1.22 | 7.95 |
| PVD-DM-CKD | 2.63 | 5.59 |
| PVD-DM-Cancer | 0.98 | 6.6 |
| PVD-CKD-Cancer | 0.83 | 7.95 |
| CVA-RESP-CKD | 0.93 | 6.58 |
| CVA-RESP-Cancer | 0.59 | 8.75 |
| CVA-DM-CKD | 1.37 | 5.52 |
| CVA-DM-Cancer | 0.54 | 7.16 |
| CVA-Hemi-Para-CKD | 0.5 | 5.28 |
| RESP-Rheum-Cancer | 0.59 | 5.82 |
| RESP-DM-Cancer | 2.38 | 6.06 |
| RESP-CKD-Cancer | 1.53 | 7.94 |
| Liver-DM-CKD | 0.53 | 5.98 |
| DM-CKD-Cancer | 1.78 | 6.43 |
| CCF-MI-PVD-RESP | 0.64 | 7.67 |
| CCF-MI-PVD-DM | 0.74 | 6.96 |
| CCF-MI-PVD-CKD | 0.7 | 8.81 |
| CCF-MI-RESP-DM | 0.92 | 5.72 |
| CCF-MI-RESP-CKD | 0.83 | 7.63 |
| CCF-MI-DM-CKD | 1.09 | 6.74 |
| CCF-PVD-RESP-DM | 0.71 | 7.67 |
| CCF-PVD-RESP-CKD | 0.75 | 9.44 |
| CCF-PVD-DM-CKD | 0.95 | 8.39 |
| CCF-RESP-DM-CKD | 1.31 | 6.95 |
| MI-PVD-RESP-DM | 0.6 | 5.64 |
| MI-PVD-DM-CKD | 0.7 | 6.75 |
| MI-RESP-DM-CKD | 0.75 | 5.85 |
| PVD-RESP-DM-CKD | 0.78 | 6.99 |
| RESP-DM-CKD-Cancer | 0.51 | 7.85 |
